# Phenotyping COVID-19 respiratory failure in spontaneously breathing patients with AI on lung CT-scan

**DOI:** 10.1186/s13054-024-05046-3

**Published:** 2024-08-05

**Authors:** Emanuele Rezoagli, Yi Xin, Davide Signori, Wenli Sun, Sarah Gerard, Kevin L. Delucchi, Aurora Magliocca, Giovanni Vitale, Matteo Giacomini, Linda Mussoni, Jonathan Montomoli, Matteo Subert, Alessandra Ponti, Savino Spadaro, Giancarla Poli, Francesco Casola, Jacob Herrmann, Giuseppe Foti, Carolyn S. Calfee, John Laffey, Giacomo Bellani, Maurizio Cereda, for the CT-COVID19 Multicenter Study Group, Ferdinando Luca Lorini, Pietro Bonaffini, Matteo Cazzaniga, Irene Ottaviani, Mario Tavola, Asia Borgo, Livio Ferraris, Filippo Serra, Stefano Gatti, Davide Ippolito, Beatrice Tamagnini, Marino Gatti, Massimo Arlotti, Emiliano Gamberini, Enrico Cavagna, Giuseppe Galbiati, Davide De Ponti

**Affiliations:** 1 School of Medicine and Surgery, University of Milano-Bicocca, Monza, Italy; 2 Department of Emergency and Intensive Care, Fondazione IRCCS San Gerardo dei Tintori Hospital, Monza, Italy; 3 Department of Anesthesiology, Critical Care, and Pain Medicine, Massachusetts General Hospital, Harvard Medical School, Boston, USA; 4 Department of Anesthesiology and Critical Care, University of Pennsylvania, Philadelphia, USA; 5 Department of Biostatistics and Epidemiology, University of Pennsylvania, Philadelphia, USA; 6 Roy J. Carver Department of Biomedical Engineering, University of Iowa, Iowa City, IA USA; 7 Department of Psychiatry and Behavioral Sciences, University of California, San Francisco, San Francisco, CA USA; 8 Department of Anesthesia and Intensive Care Medicine, Policlinico San Marco, Gruppo Ospedaliero San Donato, Bergamo, Italy; 9 Department of Medical Physiopathology and Transplants, University of Milan, Milan, Italy; 10 Istituto per la Sicurezza Sociale, San Marino, San Marino; 11 Department of Anesthesia and Intensive Care, Infermi Hospital, AUSL Romagna, Rimini, Italy; 12 Department of Anesthesia and Intensive Care Medicine, Melzo-Gorgonzola Hospital, Azienda Socio-Sanitaria Territoriale Melegnano e della Martesana, Melegnano, Milan, Italy; 13 Department of Anesthesiology and Intensive Care, ASST Lecco, Lecco, Italy; 14 Anesthesia and Intensive Care, Azienda Ospedaliero-Universitaria of Ferrara, Ferrara, Italy; 15 Department of Translational Medicine, University of Ferrara, Ferrara, Italy; 16 Department of Anaesthesia and Critical Care Medicine, Papa Giovanni XXIII Hospital, Bergamo, Italy; 17 Department of Physics, Harvard University, 17 Oxford St., Cambridge, MA 02138 USA; 18 Harvard-Smithsonian Centre for Astrophysics, 60 Garden St., Cambridge, MA 02138 USA; 19 Department of Medicine, Cardiovascular Research Institute, University of California, San Francisco, CA USA; 20 Department of Anesthesia, Cardiovascular Research Institute, University of California, San Francisco, CA USA; 21 School of Medicine, National University of Ireland Galway, Galway, Ireland; 22 Department of Anaesthesia and Intensive Care Medicine, Galway University Hospitals, Galway, Ireland; 23 University of Trento, Centre for Medical Sciences-CISMed, Trento, Italy; 24 Department of Anesthesia and Intensive Care, Santa Chiara Hospital, Trento, Italy

**Keywords:** COVID-19, Respiratory failure, Computed tomography, Artificial intelligence, Subphenotypes, Latent class analysis

## Abstract

**Background:**

Automated analysis of lung computed tomography (CT) scans may help characterize subphenotypes of acute respiratory illness. We integrated lung CT features measured via deep learning with clinical and laboratory data in spontaneously breathing subjects to enhance the identification of COVID-19 subphenotypes.

**Methods:**

This is a multicenter observational cohort study in spontaneously breathing patients with COVID-19 respiratory failure exposed to early lung CT within 7 days of admission. We explored lung CT images using deep learning approaches to quantitative and qualitative analyses; latent class analysis (LCA) by using clinical, laboratory and lung CT variables; regional differences between subphenotypes following 3D spatial trajectories.

**Results:**

Complete datasets were available in 559 patients. LCA identified two subphenotypes (subphenotype 1 and 2). As compared with subphenotype 2 (n = 403), subphenotype 1 patients (n = 156) were older, had higher inflammatory biomarkers, and were more hypoxemic. Lungs in subphenotype 1 had a higher density gravitational gradient with a greater proportion of consolidated lungs as compared with subphenotype 2. In contrast, subphenotype 2 had a higher density submantellar–hilar gradient with a greater proportion of ground glass opacities as compared with subphenotype 1. Subphenotype 1 showed higher prevalence of comorbidities associated with endothelial dysfunction and higher 90-day mortality than subphenotype 2, even after adjustment for clinically meaningful variables.

**Conclusions:**

Integrating lung-CT data in a LCA allowed us to identify two subphenotypes of COVID-19, with different clinical trajectories. These exploratory findings suggest a role of automated imaging characterization guided by machine learning in subphenotyping patients with respiratory failure.

*Trial registration*: ClinicalTrials.gov Identifier: NCT04395482. Registration date: 19/05/2020.

**Supplementary Information:**

The online version contains supplementary material available at 10.1186/s13054-024-05046-3.

## Introduction

Categorizing heterogeneous populations of critically ill patients into distinct groups has recently gained prominence because of its potential to predict outcomes. Such an approach is applicable to disparate conditions such as sepsis [1, 2], acute kidney injury [3], and acute respiratory distress syndrome (ARDS) [4]. Here, latent class analysis (LCA) showed promise in the identification of ARDS sub-phenotypes with different biologic features, treatment responses, and clinical trajectories [5–8].

Computed tomography (CT) may contribute to better stratification of the severity of acute pulmonary illness through topographic description of lung morphology. In ARDS, patient categorization by diffuse rather than focal infiltrates on lung CT was associated with higher mortality and worse respiratory mechanics [9]. However, the recent LIVE STUDY [10] was unable to show that an imaging-guided strategy of mechanical ventilation improved survival. Patient miscategorization due to heterogeneous protocols of image acquisition and subjective analysis may explain this result.

Rapid technological improvements in image processing and data modelling enable the objective characterization of lung morphology patterns for prognostic purposes [11]. Machine learning has been recently proposed to quantitatively and qualitatively evaluate large datasets of CT images. In particular, automated segmentation (i.e. separation of pulmonary from non-pulmonary tissue) by deep neural networks allows high-throughput image processing in ways that were previously impossible [12, 13]. The potential to use automatically processed CT data to predict outcomes, however, is still unexplored in acute respiratory illness.

Because of the success of LCA, using this statistical approach to integrate CT data with clinical and biological variables may enhance patient stratification in terms of severity and response to treatment. We hypothesized that, in a large population of patients with acute respiratory illness, LCA incorporating lung CT data, explored by deep neural network, may improve characterization of pathophysiology and offer a tool to triage patients, correlating radiological patterns to disease progression and treatment response. We therefore tested this hypothesis in spontaneously breathing COVID-19 patients who, being hospitalized, had a high risk of evolving to acute respiratory failure and death. The objectives of our study were to: (1) identify COVID-19 subphenotypes by incorporating pattern recognition of lung CT scans in a LCA; (2) characterize regional quantitative and qualitative lung CT data in each COVID-19 subphenotype by deep learning analysis; and (3) explore whether the severity stratification by LCA of clinical, laboratory and CT data may have an independent association with mortality.

## Methods

### Ethical consideration and data acquisition

The study was performed under the Declaration of Helsinki and in agreement with the Italian good clinical practice recommendations (D.M. Sanità del 15/07/97 e s.m.i.) and with the applied healthcare hospital protocols. No change of current clinical practice or clinical protocols in use were taken in place in the enrolled study population. Considering the retrospective nature of the proposed study, we did not anticipate risks nor benefits that might be added to the patients. Moreover, in the presence of technical difficulties related to the emergency health context to obtain an informed consent from patients in that period of pandemic, informed consent was waived. For this reason and for the great public interest of the project, the research was conducted in the context of the authorizations guaranteed by Article 89 of the GDPR EU Regulation 2016/679, which guarantees the treatment for purposes of public interest, scientific or historical research or for statistical purposes of health data. Personal data were handled in compliance with the European Regulation on the Protection of Personal Data (GDPR), the Legislative Decree 196/2003 and subsequent amendments and additions, and any other Italian law applicable to the protection of personal data (henceforth referred to as the “applicable data protection law”). Data were collected in a pseudo-anonymous way through paper case report forms, digitalized on a University of Milano-Bicocca Institutional Google drive account and analyzed by the scientific coordinator of the project (E.R.). Favorable judgment for the execution of the study was obtained before data acquisition from the local institutional review board of the coordinating center Fondazione IRCCS San Gerardo dei Tintori, Monza, Italy (Approval date: 24/04/2020; number 3375) and from the local institutional review board of each enrolled center (Policlinico San Marco, Gruppo Ospedaliero San Donato, Zingonia, Bergamo, Italy; Ospedale Infermi, Rimini, Italy; Ospedale Papa Giovanni XXIII, Bergamo, Italy; Ospedale Alessandro Manzoni, Lecco, Italy; Arcispedale Sant’Anna, Ferrara, Italy; Ospedale Santa Maria delle Stelle, Melzo, Italy; Istituto Sicureza Sociale, Repubblica di San Marino).

Baseline characteristics (age, sex, body mass index, comorbidities) and clinical illness severity (Sequential Organ Failure Assessment (SOFA) and pH) were collected, together with laboratory biomarkers, blood gas analysis, respiratory assistance, and hemodynamic data at hospital admission. Lung CT scans acquired for clinical purposes within the first week since hospital admission were obtained. Data on drug treatments and complications during hospital admissions, outcomes including length of stay (in ICU and in hospital), use of non-invasive respiratory support, mechanical ventilation-free days, limitation of life sustaining measures, ICU mortality, and hospital mortality were recorded.

### Inclusion and exclusion criteria

Inclusion criteria:

1. patients ≥ 18 years;
2. positive confirmation of SARS-CoV-2 infection with nucleic acid amplification test or serology of SARS-CoV2 by nasopharyngeal swab, broncho-aspirate sample or bronchoalveolar lavage;
3. lung CT scan performed within 7 days since hospital admission.

Exclusion criteria:

1. Patients undergoing mechanical ventilation during CT acquisition;
2. Patients with incomplete data to develop the LCA model using clinical, biological and CT data.

For the current analysis we included patients who were admitted to the Emergency Department with a clinical diagnosis of COVID-19 respiratory failure.

### Chest CT quantification

The lung CT scan images were collected and anonymized and then sent by the University of Milano-Bicocca Institutional Google drive account to the University of Pennsylvania, Department of Anesthesiology and Critical Care and the Department of Radiology (M.C., Y.X., S.G., J.H.) in a de-identified format for advanced quantitative analysis taking advantage of artificial intelligence using deep learning algorithms [14]. CT images were segmented using an established convolutional neural network (CNN) previously validated [12]. The masks included vasculature and airways inside the lungs, but excluded major airways (e.g., trachea) and vessels outside the lung lobes in the hilum area. Therefore, the role of CNN allowed to provide automated segmentations of each lung into 15 regions-of-interest (ROI) for the subsequent analysis as follows:

- whole lung;
- five individual lobes (left upper lobe (LUL), left lower lobe (LLL), right upper lobe (RUL), right middle lobe (RML), and right lower lobe (RLL));
- the analysis by the 3 axes of space (i.e. X, Y and Z) that were three equally sized (by pixel counts) including horizontal ventral-to-dorsal regions (Ventral; Dorso-Ventral; Dorsal), vertical apical-to-basal regions (Apical; Basal–Apical; Basal), and three concentric submantellar-to-hilar regions (Submantellar; Central; Hilar) [15]. After segmentation, whole-lung and lobar lung masks were inspected by a trained investigator (Y.X.), and manually adjusted using ITK-snap software [16]. For each ROI, six parameters were analyzed [17, 18]:
- average CT density in Hounsfield Units (HU);
- lung gas volume by density analysis;
- lung weight by density analysis;
- percentage of consolidated tissue (CT density > − 200 HU);
- percentage of ground glass opacity (GGO) (− 200 HU > CT density > − 750 HU); and
- percentage of total injury.

In sum, a total of ninety lung features were calculated for each patient, consisting of six parameters for each of fifteen regions. We calculated the gravitational (ventro-dorsal), the apical–basal, and the submantellar–hilar lung density gradients by linear fitting density, percentage of GGO, and percentage of consolidation in three corresponding regions. The slope of this linear fit was compared between latent classes.

### Latent class analysis

Latent class analysis (LCA) is a well-established statistical technique that employs mixture modeling to identify the most appropriate model for a data set, based on the premise that the data encompasses several unobserved groups or classes. Unlike traditional regression analyses, which aim to delineate the relationship between pre-defined independent variables and a specified outcome, LCA identifies potential subgroups within the data based on combinations of baseline variables, without necessarily linking them to an outcome.

We implemented LCA following the methodological guidelines to LCA as described by Sinha et al. [19], by amalgamating mixed clinical, laboratory, and CT data. Decision on the variables included (n = 15) in the LCA model was based on clinical illness severity at hospital admission and on previously published work [8, 20]. High correlation was explored, and the correlation matrix was plotted in online supplemental Fig. 1. The absolute value of correlations between five pairs was greater than 0.7 [(HCO_3_^−^, PaCO_2_), (Lung gas volume, GGO), (Lung gas volume, Mean lung HU), (GGO, Mean lung HU), (Consolidation, Mean lung HU)], indicating strong correlations. Therefore, mean lung HU, GGO (proportion of ground glass opacities) and HCO_3_^−^ were removed to avoid high correlation. From 559 samples, the final 12 variables (i.e. PaO_2_/FiO_2_, Lung gas volume, Temperature, PaCO_2_, Total Bilirubin, Platelets, Age, Lung mass, Creatinine, hs-CRP, WBC, Consolidation fraction) were included in the LCA model with different numbers of classes and specifications of covariance matrix structures. Depending on the model configuration, the identified classes can show different class-specific covariances [21]. We explored three settings of covariance-variance structure as shown in supplemental Fig. 2. Under the assumption of freed variance and covariances, we compared the BIC, averaged uncertainty and entropy across entire samples among 2, 3, 4, 5 and 6 classes (supplemental Table 1). The optimal model that yielded the smallest BIC and uncertainty was the one with two-classes. In addition, entropy was computed as a measure of effective separation. However, it is not a reliable sole criterion for choosing the best model because a model that overfits may also exhibit high entropy [19].

### Statistical analysis

Continuous data are reported as mean ± standard deviation (SD) or median and interquartile range (IQR). Categorical variables are expressed as proportions (frequency). Differences between the 2 clusters were assessed by unpaired Student’s T-test or U Mann–Whitney test as appropriate. Differences between categorical data were assessed by using Pearson’s chi-square test or Fisher’s exact test. Correlation between quantitative lung computed tomography data and gas exchange was assessed by linear regression analysis and Pearson correlation coefficient was reported. Differences in 90-day survival across subphenotypes was explored by Kaplan–Meier approach. Univariable and multivariable Cox proportional regression models were performed to explore the independent association of subphenotypes with 90-day mortality by including clinically meaningful covariates. Mortality risk was reported by hazard ratio with 95% confidence interval. Clinically meaningful covariates were decided a priori to adjust the multivariable models as follows: sex, the presence of any comorbidities, the decision of limitation of life sustaining measures. Adjusted models were ranked by their Akaike information criterion (AIC) and their Bayesian information criterion (BIC). AIC and BIC address both goodness-of-fit and simplicity of a model. Since we compared models with the same number of independent variables for the same set of patients, the lowest AIC and BIC represented the best fit model. Statistical significance was considered with a *p* < 0.05 (two-tailed). Further, we investigated LCA modeling by only including clinical and laboratory data (i.e. PaO_2_/FiO_2_, Temperature, PaCO_2_, Total Bilirubin, Platelets, Age, Creatinine, hs-CRP, WBC) or only including CT derived features (i.e. Lung gas volume, Lung mass, Consolidation fraction) to assess whether the most complete LCA model including overall mixed clinical, laboratory, and CT data showed a better association with 90-day mortality and the highest goodness of model fitting. Statistical analysis was performed by SPSS software v28 (IBM Corp., Armonk, NY, USA), R-project (Version 4.3.2) and Stata/MP 17.0 (Copyright 1985-2021 StataCorp LLC (College Station, TX, 77845, USA).

### Sample size

We aimed to collect data from 500 patients at least, as this is considered an adequate sample size to conduct LCA [19].

Comprehensive information on methods is reported in the Supplemental material.

This study followed The Strengthening the Reporting of Observational studies in Epidemiology (STROBE) reporting guideline checklist.

## Results

### Patient population and stratification by LCA

Clinical, laboratory and CT data were collected between February and April 2020, during the peak of the first Italian wave of the COVID-19 pandemic.

Out of 853 patients, 810 fulfilled study inclusion criteria and had a diagnosis of COVID-19 respiratory failure at the ED admission. Five-hundred and fifty-nine (559) patients had complete records including clinical, laboratory and CT variables to build the LCA model (online supplemental Fig. 3). Patients’ characteristics are reported in Table 1.

**Table 1:** Baseline characteristics, comorbidities, clinical illness severity, respiratory support at hospital admission; treatments and outcomes of patients stratified by subphenotypes

	Overall	Subphenotype 1	Subphenotype 2	*p* value
*Reason of hospital admission*				
Respiratory failure, n (%)	559 (100)	156 (28)	403 (72)	–
*Baseline characteristics*				
BMI, kg/m^2^; mean (standard deviation), N = 171	27.7 (4.6)	27.4 (5.2)	27.8 (4.3)	0.572
Sex, F (%)	196 (35)	58 (37)	138 (34)	0.514
Time between admission and CT scan, days; median (IQR)	0 (0;0)	0 (0;0)	0 (0;0)	0.138
*Comorbidities*				
COPD, n (%)	28 (5)	12 (8)	16 (4)	0.070
Asthma, n (%)	27 (5)	7 (5)	20 (5)	0.814
Congestive heart failure, n (%)	26 (5)	12 (8)	14 (4)	0.034
Chronic kidney disease, n (%)	37 (7)	31 (20)	6 (2)	< 0.001
Chronic liver failure, n (%)	3 (1)	0 (0)	3 (1)	0.280
Solid cancer, n (%)	14 (3)	6 (4)	8 (2)	0.207
Hematologic malignancy, n (%)	9 (2)	1 (1)	8 (2)	0.257
Immune mediated disease, n (%)	20 (4)	4 (3)	16 (4)	0.422
Diabetes, n (%)	97 (17)	39 (25)	58 (14)	0.003
Systemic hypertension, n (%)	284 (51)	99 (63)	185 (46)	< 0.001
Antihypertensive drugs, n (%) N = 549				
ACE-inhibitors	346 (63)	81 (53)	256 (67)	0.004
ARBs	90 (16)	27 (18)	63 (16)	
Others/unknown	113 (21)	46 (30)	67 (17)	
Pulmonary hypertension, n (%)	3 (1)	3 (2)	0 (0)	0.005
Atrial fibrillation, n (%)	57 (10)	22 (14)	35 (9)	0.058
OSAS, n (%)	6 (1)	2 (1)	4 (1)	0.766
cPAP at home, n (%)	4 (1)	1 (1)	3 (1)	0.896
Pacing, n (%)	10 (2)	6 (4)	4 (1)	0.022
Any comorbidities, n (%)	404 (72)	128 (82)	276 (69)	0.001
*Clinical illness severity*				
pH, mean (standard deviation)	7.47 (0.05)	7.45 (0.06)	7.47 (0.04)	< 0.001
PaO_2_/FiO_2_, mean (standard deviation), mmHg	252 (103)	188 (95)	277 (95)	< 0.001
HCO_3_^−^, mean (standard deviation), mEq/L	23 (4)	23 (4)	23 (3)	0.168
D-dimer, mean (standard deviation), mg/L, n = 287	2.7 (5.9)	4.5 (7.8)	1.8 (4.6)	< 0.001
*Respiratory support, N = 559*				
Oxygen delivery, n (%)				
Room air	367 (66)	61 (39)	306 (76)	< 0.001
LFO	189 (34)	93 (60)	96 (24)	
Non-invasive ventilation, n (%)				
cPAP	2 (0)	1 (1)	1 (0)	
*Pharmacological treatments*				
Antivirals; N = 557	202 (36)	47 (30)	155 (39)	0.060
Antibiotic treatment; N = 556	491 (88)	149 (96)	342 (86)	< 0.001
Steroids; N = 553	128 (23)	39 (25)	89 (22)	0.483
Hydroxycloroquine; N = 555	406 (73)	131 (85)	275 (69)	< 0.001
Cloroquine phosphate; N = 554	48 (9)	1 (1)	47 (12)	< 0.001
Immunoglobulins IV; N = 553	2 (0)	0 (0)	2 (1)	0.377
Tocilizumab; N = 553	30 (5)	9 (6)	21 (5)	0.805
Anakinra; N = 552	4 (1)	1 (1)	3 (1)	0.897
Thrombolysis; N = 553	2 (0)	2 (1)	0 (0)	0.023
Anticoagulation; N = 552	379 (69)	135 (87)	244 (62)	< 0.001
Anticoagulant; N = 550				
None	149 (27)	20 (13)	129 (33)	< 0.001
LMWH	371 (68)	128 (82)	243 (62)	
UFH	2 (0)	1 (1)	1 (0)	
Argatroban	16 (3)	4 (3)	12 (3)	
Fondaparinux	2 (0)	0 (0)	2 (1)	
Salicilic acid	10 (2)	3 (2)	7 (2)	
*Organ support techniques*				
CRRT; N = 553	9 (2)	5 (3)	4 (1)	0.064
*Complications*				
All Bacterial overinfections; N = 556	57 (10)	28 (18)	29 (7)	< 0.001
Lung overinfection; N = 556	33 (6)	18 (12)	15 (4)	< 0.001
Blood overinfection; N = 555	17 (3)	5 (3)	12 (3)	0.890
Urinary tract overinfection; N = 555	9 (2)	3 (2)	6 (2)	0.716
Soft tissues overinfection; N = 555	4 (1)	1 (1)	3 (1)	0.896
Abdominal overinfection; N = 555	2 (0)	1 (1)	1 (0)	0.486
Stroke; N = 552	6 (1)	2 (1)	4 (1)	0.773
Venous thromboembolism; N = 552	7 (1)	3 (2)	4 (1)	0.381
Pulmonary thromboembolism; N = 552	9 (2)	6 (4)	3 (1)	0.009
Tracheostomy	7 (1)	2 (1)	5 (1)	0.969
*Outcomes*				
MV duration (days); N = 49	14 (12)	15 (15)	13 (11)	0.544
Limitation of life sustaining measures; N = 549	132 (24)	71 (46)	61 (15)	< 0.001
ICU admission—N = 59	59 (11)	22 (14)	37 (9)	0.089
ICU mortality—N = 59	35 (57)	15 (63)	20 (54)	0.515
ICU LOS; N = 57	17 (14)	16 (14)	18 (14)	0.639
Survivors; N = 23	22 (16)	20 (22)	23 (14)	0.794
Dead; N = 34	14 (10)	14 (8)	14 (12)	0.997
Hospital mortality	180 (32)	91 (58)	89 (22)	< 0.001
Hospital LOS; N = 554	13 (15)	14 (13)	13 (15)	0.305
Survivors; N = 371	14 (16)	21 (15)	13 (16)	< 0.001
Dead; N = 180	10 (10)	9 (7)	12 (11)	0.093

Differences between the 2 subphenotypes were assessed and reported in *p* value column. Continuous data are expressed as mean (standard deviation), categorical variables as count (relative frequency %). In the presence of missing data, sample size was reported

*ACE* angiotensin converting enzyme, *ARB* angiotensin receptor blockers, *BMI* body mass index, *COPD* chronic obstructive pulmonary disease, *cPAP* continuous positive airway pressure, *ICU* intensive care unit, *LFO* low-flow oxygen, *LMWH* low molecular weight heparin, *LOS* length of stay, *OSAS* obstructive sleep apnea syndrome, *UFH* unfractionated heparin

**Fig. 1:**
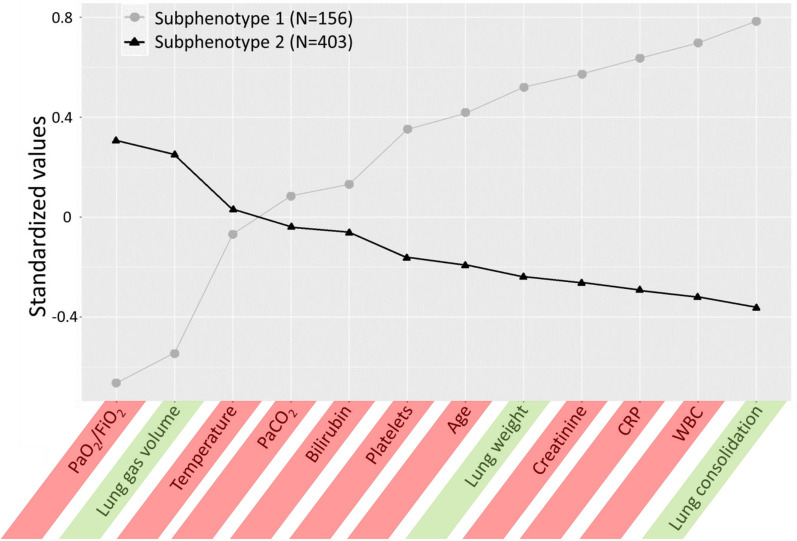
Differences in standardized values of each continuous variable by LCA derived subphenotypes. The variables are sorted based on the degree of separation between the subphenotypes, from maximum positive separation on the left (i.e., subphenotype 2 higher than subphenotype 1) to maximum negative separation on the right (i.e., subphenotype 2 lower than subphenotype 1). The y-axis describes the standardized variable values, in which all means are scaled to zero and standard deviations (SDs) to one. A value of + 1 for the investigated standardized variable means that the mean value for a given subphenotype was one SD higher than the mean value in the cohort as a whole. Mean values are joined by lines to facilitate displaying subphenotype profiles. Variables included to investigate LCA derived subphenotypes are highlighted in green (CT-derived features) and red (clinical and laboratory parameters). *WBC* white blood cells, *CRP* C-reactive protein, *PaCO*_*2*_ arterial carbon dioxide partial pressure, *PaO*_*2*_*/FiO*_*2*_ ratio of arterial oxygen partial pressure to fractional inspired oxygen

We identified 2 different clusters of patients that we labeled as subphenotype 1 and subphenotype 2 subphenotypes (Fig. 1). Differences in LCA variables are reported in online supplemental Table 2. The subphenotype 1 was radiologically characterized by higher lung weight, lower lung gas volume, and higher proportion of consolidation. Oxygenation was worse in the subphenotype 1 as compared with the subphenotype 2, with no difference in pCO_2_ levels. Inflammatory biomarkers such as white blood cells (WBC), high sensitivity C-reactive protein, and platelets were higher in the subphenotype 1. Patients in the subphenotype 1 were older and had higher creatinine levels as compared with the subphenotype 2. Comorbidities associated with endothelial dysfunction (e.g. systemic hypertension, diabetes, chronic kidney disease and congestive heart failure) were more prominent in subphenotype 1. Consistently, more endothelial activation was observed by higher levels of D-Dimers in subphenotype 1. Low-flow FiO_2_ requirement was higher in the subphenotype 1 at hospital admission (Table 1).

### Quantitative and qualitative CT analysis by automated segmentation using deep neural network algorithm

**Fig. 2:**
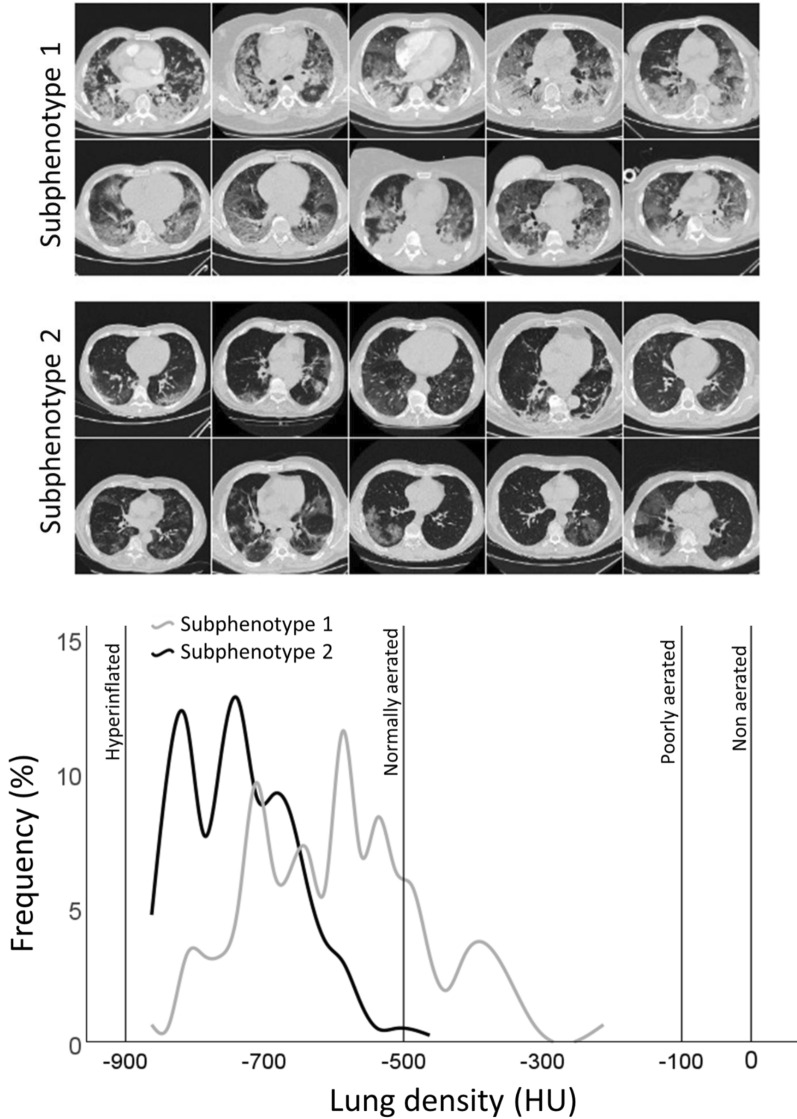
Ten representative images of lung CT scan images in the subphenotype 1 (upper panel) and subphenotype 2 (middle panel). In the lower panel, different lung cumulative density distribution measured with CT X-rays attenuation of the whole lung between the two subphenotypes. Interpolation lines are displayed to reduce frequency oscillation. Mean lung density < − 900 HU: Hyperinflated; − 900 HU ≤ Mean lung density < − 500 HU: normally aerated; − 500 HU ≤ Mean lung density < − 100 HU: poorly aerated; Mean lung density ≥ − 100 HU ≤ 0 HU: non aerated. *HU* hounsfield units

Exemplary images of 10 patients with subphenotype 1 (Fig. 2 upper panel) and subphenotype 2 (Fig. 2 middle panel) are shown. Frequency distribution of overall mean lung density in the 2 different subphenotypes is reported in Fig. 2 bottom panel. We provided a detailed description of regional quantitative and qualitative CT differences across the 2 latent classes in Table 2.

**Table 2:** Regional quantitative and qualitative analysis of lung CT images stratified by subphenotypes

	Overall (N = 559)	Subphenotype 1 (N = 156)	Subphenotype 2 (N = 403)	*p* value
				
*Whole lung*				
Mean lung density (HU)	− 702 (106)	− 613 (112)	− 736 (80)	< 0.001
Lung gas volume (L)	2.91 (1.25)	2.16 (0.9)	3.2 (1.25)	< 0.001
Lung weight (kg)	1.12 (0.33)	1.28 (0.42)	1.05 (0.27)	< 0.001
Total injury (fraction)	0.43 (0.19)	0.57 (0.17)	0.37 (0.17)	< 0.001
GGO (fraction)	0.36 (0.16)	0.45 (0.14)	0.33 (0.15)	< 0.001
Consolidation (fraction)	0.07 (0.06)	0.12 (0.09)	0.05 (0.03)	< 0.001
				
*Left upper lobe*				
Mean lung density (HU)	− 734 (110)	− 650 (135)	− 767 (77)	< 0.001
Lung gas volume (L)	0.8 (0.36)	0.62 (0.28)	0.87 (0.36)	< 0.001
Lung weight (kg)	0.26 (0.09)	0.3 (0.1)	0.24 (0.08)	< 0.001
Total injury (fraction)	0.38 (0.2)	0.52 (0.21)	0.32 (0.17)	< 0.001
GGO (fraction)	0.33 (0.17)	0.43 (0.16)	0.29 (0.16)	< 0.001
Consolidation (fraction)	0.05 (0.07)	0.09 (0.11)	0.03 (0.02)	< 0.001
*Left lower lobe*				
Mean lung density (HU)	− 647 (145)	− 537 (169)	− 689 (109)	< 0.001
Lung gas volume (L)	0.55 (0.32)	0.38 (0.23)	0.62 (0.33)	< 0.001
Lung weight (kg)	0.26 (0.09)	0.29 (0.12)	0.24 (0.07)	< 0.001
Total injury (fraction)	0.52 (0.23)	0.67 (0.2)	0.46 (0.21)	< 0.001
GGO (fraction)	0.42 (0.18)	0.49 (0.16)	0.39 (0.18)	< 0.001
Consolidation (fraction)	0.1 (0.11)	0.18 (0.17)	0.07 (0.06)	< 0.001
*Right upper lobe*				
Mean lung density (HU)	− 723 (113)	− 642 (129)	− 755 (88)	< 0.001
Lung gas volume (L)	0.64 (0.29)	0.51 (0.25)	0.69 (0.29)	< 0.001
Lung weight (kg)	0.22 (0.08)	0.26 (0.09)	0.2 (0.07)	< 0.001
Total injury (fraction)	0.39 (0.21)	0.53 (0.21)	0.34 (0.19)	< 0.001
GGO (fraction)	0.34 (0.18)	0.43 (0.16)	0.3 (0.17)	< 0.001
Consolidation (fraction)	0.06 (0.06)	0.1 (0.1)	0.04 (0.03)	< 0.001
*Right medium lobe*				
Mean lung density (HU)	− 758 (92)	− 694 (99)	− 783 (76)	< 0.001
Lung gas volume (L)	0.32 (0.15)	0.25 (0.12)	0.34 (0.15)	< 0.001
Lung weight (kg)	0.09 (0.03)	0.1 (0.04)	0.09 (0.03)	< 0.001
Total injury (fraction)	0.34 (0.19)	0.47 (0.19)	0.29 (0.17)	< 0.001
GGO (fraction)	0.3 (0.17)	0.41 (0.16)	0.26 (0.16)	< 0.001
Consolidation (fraction)	0.04 (0.03)	0.06 (0.05)	0.03 (0.02)	< 0.001
*Right lower lobe*				
Mean lung density (HU)	− 635 (146)	− 516 (158)	− 682 (112)	< 0.001
Lung gas volume (L)	0.6 (0.34)	0.4 (0.24)	0.67 (0.34)	< 0.001
Lung weight (kg)	0.29 (0.11)	0.34 (0.15)	0.27 (0.08)	< 0.001
Total injury (fraction)	0.53 (0.23)	0.7 (0.18)	0.47 (0.22)	< 0.001
GGO (fraction)	0.42 (0.18)	0.5 (0.15)	0.39 (0.18)	< 0.001
Consolidation (fraction)	0.11 (0.11)	0.19 (0.16)	0.07 (0.05)	< 0.001
				
*Basal*				
Mean lung density (HU)	− 679 (121)	− 585 (124)	− 715 (98)	< 0.001
Lung gas volume (L)	0.93 (0.43)	0.68 (0.3)	1.03 (0.43)	< 0.001
Lung weight (kg)	0.39 (0.12)	0.45 (0.15)	0.37 (0.1)	< 0.001
Total injury (fraction)	0.47 (0.21)	0.62 (0.18)	0.42 (0.2)	< 0.001
GGO (fraction)	0.4 (0.17)	0.48 (0.15)	0.36 (0.17)	< 0.001
Consolidation (fraction)	0.08 (0.07)	0.14 (0.1)	0.05 (0.04)	< 0.001
*Basal–apical*				
Mean lung density (HU)	− 695 (109)	− 604 (115)	− 730 (83)	< 0.001
Lung gas volume (L)	0.96 (0.41)	0.71 (0.3)	1.06 (0.41)	< 0.001
Lung weight (kg)	0.38 (0.12)	0.44 (0.15)	0.36 (0.1)	< 0.001
Total injury (fraction)	0.44 (0.2)	0.58 (0.18)	0.38 (0.18)	< 0.001
GGO (fraction)	0.36 (0.16)	0.45 (0.14)	0.33 (0.16)	< 0.001
Consolidation (fraction)	0.07 (0.06)	0.13 (0.09)	0.05 (0.03)	< 0.001
*Apical*				
Mean lung density (HU)	− 732 (106)	− 650 (123)	− 763 (78)	< 0.001
Lung gas volume (L)	1.02 (0.42)	0.77 (0.32)	1.11 (0.42)	< 0.001
Lung weight (kg)	0.34 (0.12)	0.39 (0.15)	0.32 (0.1)	< 0.001
Total injury (fraction)	0.38 (0.2)	0.52 (0.2)	0.32 (0.17)	< 0.001
GGO (fraction)	0.33 (0.16)	0.42 (0.15)	0.29 (0.15)	< 0.001
Consolidation (fraction)	0.05 (0.06)	0.1 (0.09)	0.03 (0.03)	< 0.001
				
*Dorsal*				
Mean lung density (HU)	− 634 (144)	− 514 (157)	− 680 (109)	< 0.001
Lung gas volume (L)	0.88 (0.43)	0.6 (0.31)	0.98 (0.42)	< 0.001
Lung weight (kg)	0.45 (0.16)	0.53 (0.2)	0.42 (0.13)	< 0.001
Total injury (fraction)	0.54 (0.23)	0.7 (0.18)	0.48 (0.21)	< 0.001
GGO (fraction)	0.43 (0.18)	0.5 (0.15)	0.41 (0.18)	< 0.001
Consolidation (fraction)	0.11 (0.11)	0.2 (0.16)	0.07 (0.05)	< 0.001
*Dorso-ventral*				
Mean lung density (HU)	− 699 (107)	− 611 (118)	− 733 (81)	< 0.001
Lung gas volume (L)	0.97 (0.41)	0.72 (0.3)	1.06 (0.41)	< 0.001
Lung weight (kg)	0.38 (0.11)	0.43 (0.14)	0.36 (0.09)	< 0.001
Total injury (fraction)	0.43 (0.2)	0.58 (0.19)	0.37 (0.18)	< 0.001
GGO (fraction)	0.36 (0.17)	0.47 (0.15)	0.33 (0.16)	< 0.001
Consolidation (fraction)	0.07 (0.06)	0.11 (0.08)	0.05 (0.03)	< 0.001
*Ventral*				
Mean lung density (HU)	− 773 (86)	− 712 (98)	− 796 (67)	< 0.001
Lung gas volume (L)	1.06 (0.42)	0.84 (0.32)	1.15 (0.42)	< 0.001
Lung weight (kg)	0.29 (0.09)	0.32 (0.12)	0.27 (0.08)	< 0.001
Total injury (fraction)	0.31 (0.18)	0.43 (0.19)	0.27 (0.15)	< 0.001
GGO (fraction)	0.29 (0.16)	0.38 (0.16)	0.25 (0.14)	< 0.001
Consolidation (fraction)	0.03 (0.03)	0.05 (0.05)	0.02 (0.01)	< 0.001
				
*Submantellar*				
Mean lung density (HU)	− 686 (104)	− 600 (107)	− 719 (82)	< 0.001
Lung gas volume (L)	1.07 (0.51)	0.83 (0.31)	1.17 (0.55)	< 0.001
Lung weight (kg)	0.46 (0.17)	0.53 (0.17)	0.44 (0.16)	< 0.001
Total injury (fraction)	0.47 (0.18)	0.6 (0.16)	0.42 (0.17)	< 0.001
GGO (fraction)	0.4 (0.15)	0.47 (0.13)	0.37 (0.15)	< 0.001
Consolidation (fraction)	0.07 (0.06)	0.12 (0.08)	0.05 (0.03)	< 0.001
*Central*				
Mean lung density (HU)	− 715 (104)	− 630 (113)	− 747 (78)	< 0.001
Lung gas volume (L)	0.78 (0.29)	0.6 (0.24)	0.85 (0.28)	< 0.001
Lung weight (kg)	0.29 (0.1)	0.34 (0.12)	0.27 (0.08)	< 0.001
Total injury (fraction)	0.4 (0.19)	0.54 (0.18)	0.35 (0.17)	< 0.001
GGO (fraction)	0.34 (0.16)	0.43 (0.14)	0.31 (0.15)	< 0.001
Consolidation (fraction)	0.06 (0.06)	0.11 (0.09)	0.04 (0.03)	< 0.001
*Hilar*				
Mean lung density (HU)	− 712 (113)	− 618 (127)	− 748 (83)	< 0.001
Lung gas volume (L)	1.06 (0.67)	0.72 (0.46)	1.19 (0.7)	< 0.001
Lung weight (kg)	0.36 (0.16)	0.4 (0.2)	0.35 (0.13)	< 0.001
Total injury (fraction)	0.4 (0.21)	0.56 (0.2)	0.34 (0.19)	< 0.001
GGO (fraction)	0.34 (0.17)	0.44 (0.16)	0.3 (0.17)	< 0.001
Consolidation (fraction)	0.06 (0.07)	0.12 (0.1)	0.04 (0.03)	< 0.001

Values are expressed as mean (standard deviation)

*HU* hounsfield units, *GGO* ground-glass opacities

**Fig. 3:**
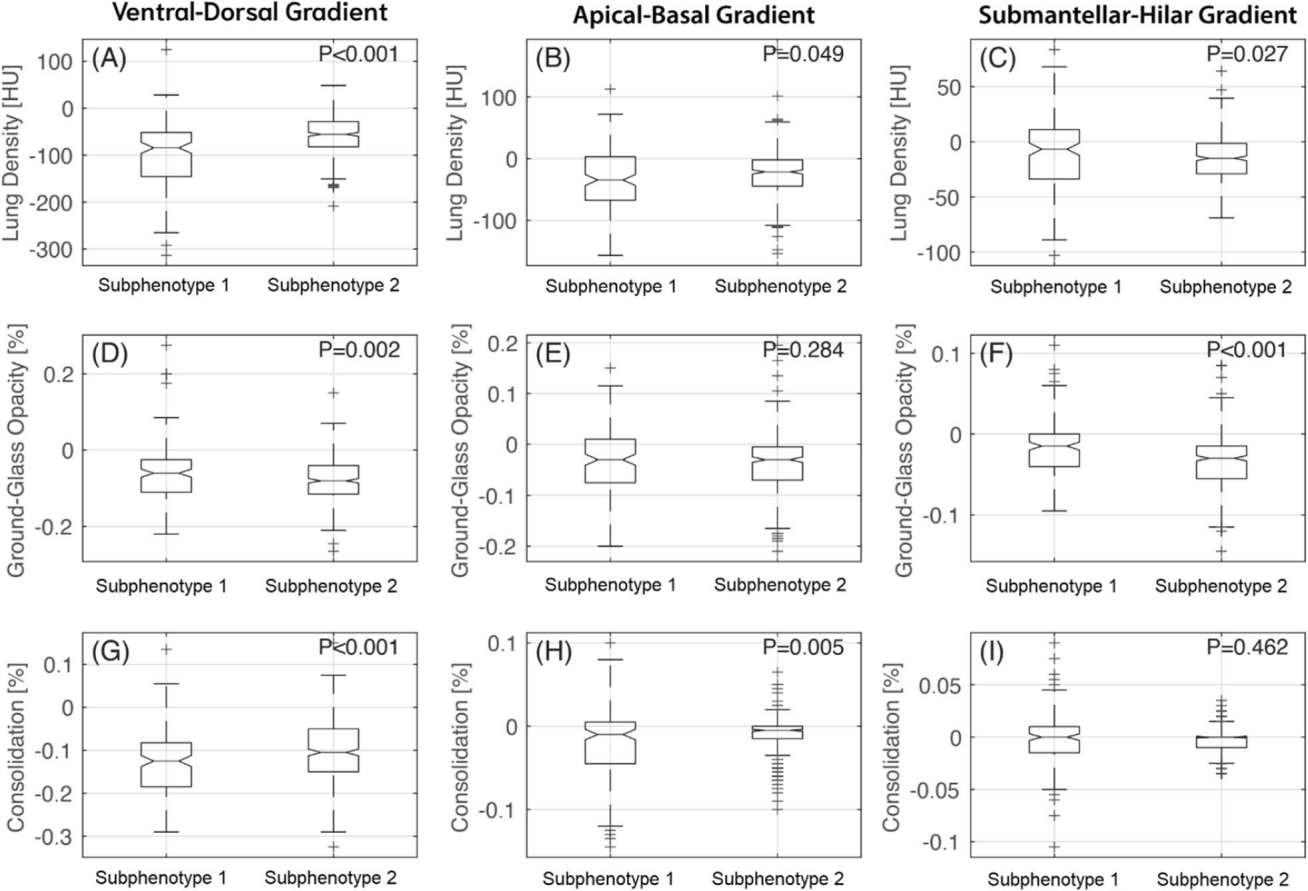
Box and whisker plots of mean lung density, ground glass opacities, and consolidation distribution in subphenotype 1 and subphenotype 2 across 3 different gradients of lung injury. Ventro-dorsal gradient (panel **A**, **D** and **G**); apical–basal gradient (panel **B**, **E** and **H**); and submantellar–hilar gradient (panel **C**, **F** and **I**)

At a regional level, the gravitational and apical–basal density gradients were significantly higher in the subphenotype 1 as compared with the subphenotype 2 (Fig. 3A, B). This was explained by a higher change in consolidation (Fig. 3G, H) and a lower change in ground glass opacities (Fig. 3D), respectively.

In contrast, the submantellar–hilar density gradient was higher in the subphenotype 2 as compared with the subphenotype 1 (Fig. 3C). This was driven by a higher change in ground glass opacities in subphenotype 2 versus subphenotype 1 (Fig. 3F).

**Fig. 4:**
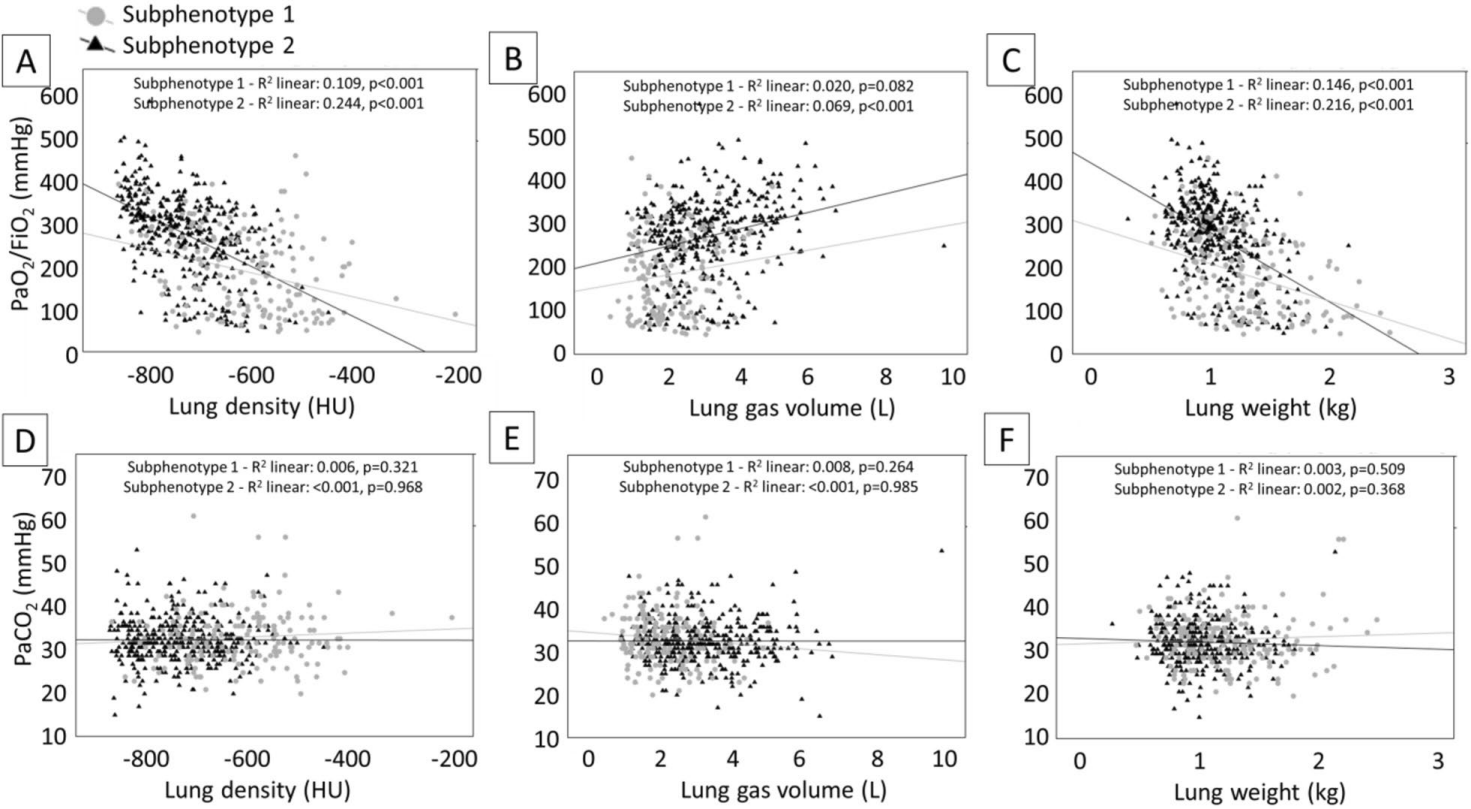
Correlation between CT derived parameters and gas exchange. Panel **A**: correlation between mean lung density and PaO_2_/FiO_2_; panel **B**: correlation between lung gas volume and PaO_2_/FiO_2_; panel **C**: correlation between lung weight and PaO_2_/FiO_2_; panel **D**: correlation between mean lung density and PaCO_2_; panel **E**: correlation between lung gas volume and PaCO_2_; panel **F**: correlation between lung weight and PaCO_2_. *HU* hounsfield units, *PaO*_*2*_*/FiO*_*2*_ ratio of arterial oxygen partial pressure to fractional inspired oxygen, *PaCO*_*2*_ arterial carbon dioxide partial pressure

Lung gas volume mildly correlated with oxygenation in patients with subphenotype 1, while mean lung density and lung weight correlated with oxygenation in both LCA clusters (Fig. 4A–C). None of the imaging features of the 2 subphenotypes correlated with PaCO_2_ levels (Fig. 4D–F).

### Pharmacological treatments and complications

During the hospital stay, the subphenotype 1 received a higher proportion of antibiotics, hydroxycloroquine and cloroquine, anticoagulation and a higher trend of steroids.

A higher rate of complications were present in subphenotype 1 as compared with subphenotype 2. (Table 1).

### Outcome analysis

The subphenotype 1 had a higher hospital mortality rate (58% vs. 22%, *p* < 0.001) and a longer length of stay (21 (15) vs. 13 (16), *p* < 0.001) in survivors—as compared with the subphenotype 2. No differences between ICU outcomes were observed. Limitation of life sustaining measure was more frequent in the subphenotype 1 (Table 1).

**Fig. 5:**
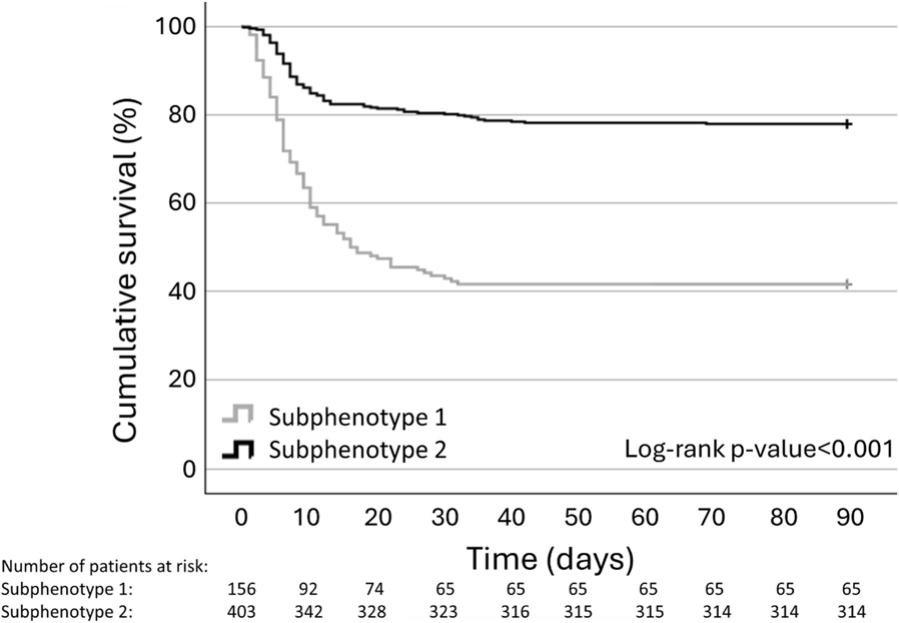
Survival at 90-day follow-up by Kaplan–Meier curves in the 2 different classes of subphenotypes

Ninety-day survival confirmed a significant difference between the 2 classes (subphenotype 1 vs. subphenotype 2, 42% vs. 78%, log-rank *p* < 0.001) (Fig. 5).

In univariable Cox proportional regression modeling, subphenotype 1 was significantly associated with 90-day mortality (HR 3.49, 95% CI [2.60–4.69], *p* < 0.001). This was confirmed after the adjustments with clinically meaningful variables including sex, the presence of any comorbidities and limitation of life sustaining measures. The highest prediction models included the one adjusted for all the tested variables (lowest AIC = 1797) and the model adjusted for both limitation of life sustaining measures and the presence of any comorbidities (lowest BIC = 1813) (Table 3).

**Table 3:** Univariable and multivariable Cox proportional regression models explore the independent association of subphenotypes with 90-day mortality by including clinically meaningful covariates

	HR (95% CI)	*p* value	AIC	BIC
*Univariable*				
Sex (Ref. M)	0.57 (0.41–0.81)	0.001	2175	2180
Any comorbidities (Ref. None)	3.26 (2.15–4.94)	< 0.001	2146	2151
Limitation of life sustaining measures (Ref. No)	28.73 (19.89–41.49)	< 0.001	1812	1816
Subphenotype 1 (Ref. Subphenotype 2)	3.49 (2.60–4.69)	< 0.001	2121	2125
*Multivariable*				
Subphenotype 1 (adjusted for sex) (Ref. Subphenotype 2)	3.57 (2.65–4.79)	< 0.001	2110	2118
Subphenotype 1 (adjusted for any comorbidities) (Ref. Subphenotype 2)	3.07 (2.28–4.13)	< 0.001	2096	2105
Subphenotype 1 (adjusted for limitation of life sustaining measures) (Ref. Subphenotype 2)	1.60 (1.17–2.18)	0.003	1805	1814
Subphenotype 1 (adjusted for sex and any comorbidities) (Ref. Subphenotype 2)	3.12 (2.32–4.21)	< 0.001	2086	2099
Subphenotype 1 (adjusted for sex and limitation of life sustaining measures) (Ref. Subphenotype 2)	1.68 (1.23–2.30)	0.001	1803	1816
Subphenotype 1 (adjusted for any comorbidities and limitation of life sustaining measures) (Ref. Subphenotype 2)	1.54 (1.13–2.10)	0.006	1800	1813
Subphenotype 1 (adjusted for sex, any comorbidities and limitation of life sustaining measures) (Ref. Subphenotype 2)	1.63 (1.19–2.23)	0.002	1797	1815

Mortality risk was reported by hazard ratio with 95% confidence interval. Adjusted models were ranked by their Akaike information criterion (AIC) and their Bayesian information criterion (BIC). N = 549

Further, we investigated whether the association between subphenotypes obtained by LCA only including clinical and laboratory data or only including CT derived features (data not shown) were differently associated with 90-day mortality. The subphenotype 1 obtained by LCA including all clinical, laboratory and CT derived variables was associated with the highest 90-day mortality risk (n = 559; subphenotype 1 versus subphenotype 2; HR 3.46; 95% CI 2.58–4.64; *p* < 0.001) and highest goodness of fit (AIC, 2153; BIC, 2157) as compared to LCA modeling only including clinical and laboratory data (n = 559; subphenotype 1 vs. subphenotype 2; HR 3.23; 95% CI 2.40–4.35; *p* < 0.001; AIC, 2164; BIC, 2169) or only including CT derived features (n = 559; subphenotype 1 vs. subphenotype 2; HR 3.13; 95% CI 2.28–4.31; *p* < 0.001; AIC, 2163; BIC, 2168).

Furthermore, as age is an important predictor of mortality and may influence clinical decision making, we explored whether retaining or removing age from the LCA may help to improve the outcome prediction in our study population. Presence (n = 559; subphenotype 1 versus subphenotype 2; HR 3.46; 95% CI 2.58–4.63; *p* < 0.001; AIC = 2153; BIC = 2157) or absence (n = 559; subphenotype 1 versus subphenotype 2; HR 3.54; 95% CI 2.64–4.75; *p* < 0.001; AIC = 2152; BIC = 2156) of age within the LCA modeling did not make difference in the prediction of 90-day mortality, as shown by AIC and BIC values. This confirmed the goodness of our original LCA modeling including age in separating latent classes independently from outcomes.

### Differences between patients included and excluded from the LCA model

A comprehensive description of differences between demographics, clinical, CT and outcome characteristics between patients with complete and incomplete data was presented in online supplemental Tables 3–5.

## Discussion

In this retrospective multicenter observational study performed during the peak of the COVID-19 pandemic in Italy, we observed the following major findings in spontaneously breathing patients during their early hospital admission:

- LCA separated two different subphenotypes using clinical, laboratory and chest CT data analyzed by AI, that were characterized by different levels of systemic inflammatory biomarkers, oxygenation, and lung injury distribution;
- using automated segmentation with deep learning analysis, we observed higher mean lung density and lower gas content in the lungs of patients within the subphenotype 1, larger proportion of consolidation and ground glass attenuation as compared with the subphenotype 2;
- the 2 subphenotypes showed different spatial heterogeneity, with a higher gravitational and apical–basal density gradient mainly led by consolidation in subphenotype 1, while a higher submantellar–hilar density gradient mainly led by ground-glass opacities in subphenotype 2;
- the subphenotype 1 had higher rate of hospital mortality, confirmed in multivariable models adjusted for clinically meaningful variables.

The SARS-CoV-2 pandemic nearly overwhelmed the Italian healthcare system in the first half of 2020, imposing a dramatic burden on intensive care units [22]. Nevertheless, this surge allowed us to collect a large amount of data on this specific respiratory condition [23, 24]. We therefore decided to perform this exploratory study to test the hypothesis that integrating radiological, clinical, and laboratory data may allow categorization of individual patients in distinct subphenotypes of acute respiratory illness.

Population enrichment by ARDS phenotyping has been proposed to reduce between-subject heterogeneity paving the road to precision medicine [25]. Within this context, the use of LCA using clinical and biological data identified an hyperinflammatory cluster of ARDS that was associated with a high mortality rate [20, 26] and differential treatment responses [8]. In contrast, the efficacy of this approach in COVID-19 respiratory failure is uncertain. Several prognostic models have been proposed for COVID-19 but did not show accurate prediction of clinical deterioration or mortality [27, 28]. Sinha et al. reported that the role of inflammation may be less impactful on outcomes than in classical ARDS [29]. Bos et al. did not report the presence of consistent respiratory subphenotypes in COVID-19 patients [30]. In contrast, Ranjeva et al. observed 2 distinct subphenotypes of COVID-19 respiratory failure with substantial differences in biochemical profiles and coagulopathy [31]. Furthermore, when using only CT data to stratify COVID-19 respiratory failure, Robba et al. reported that specific chest CT-patterns may help to optimize the ventilator strategy [32].

Filippini et al. previously applied a LCA analysis to lung-CT and ventilatory data in a small sample of mechanically ventilated patients to identify lung recruitability [33]. In contrast, we studied only patients who were captured early in their clinical course, shortly after hospital admission and while breathing spontaneously. In such population, we explored LCA by combining clinical and biological data with imaging metrics. We identified a subphenotype 1, associated with more heterogeneous injury on pulmonary CT and with the presence of higher levels of systemic inflammatory biomarkers. This subphenotype 1 had worse oxygenation, which was related to metrics of radiological severity. Moreover, our data suggest the presence of more severe vascular endothelial dysfunction in the subphenotype 1, because of the higher frequency of vascular comorbidities (e.g. diabetes, systemic hypertension, chronic renal failure and congestive cardiac failure) [34]. Higher D-Dimer levels also support endothelial dysfunction in this subgroup of patients, which is a known proxy of pulmonary hypoperfusion in COVID-19 patients [35] and may have contributed to worse gas exchange. Notably, unlike in studies of ARDS phenotypes, plasma bicarbonate did not differ between the 2 subphenotypes [5, 20, 29].

The use of machine learning techniques enables processing a large-volume image dataset, using a validated method of radiological processing [12, 13]. This quantitative lung CT analysis informed us on mean lung density distributions in both subphenotypes. We observed significant differences in mean lung density distributions, although the amount of poorly aerated lung tissue was relatively low in the subphenotype 2 and the majority of segmented lung was contained within the normal range of aeration [17]. Despite these subtle alterations in lung aeration—which may also be overemphasized by the presence of spontaneous breathing in all patients—all evaluated lung regions in the subphenotype 1 were quantitatively denser and heavier and the whole lung gas volume was lower, as compared with the subphenotype 2. Furthermore, the subphenotype 1 showed a higher quantitative gravitational and apical–basal density gradient, while the subphenotype 2 showed a higher submantellar–hilar gradient. These findings provide a morphological description of the 2 subphenotypes by adding a morphological quantification to LCA characterization of clinical severity.

The identification of two different clusters was highly prognostic, as the subphenotypes had different association with hospital mortality. We adjusted the model for clinically meaningful variables known to impact on mortality in patients with respiratory failure: sex [36], comorbidities, and limitation of life sustaining measures [37]. After adjustment, subphenotype 1 remained a robust predictor of death with an OR of 2.86 as compared with the subphenotype 2. These findings confirm a correlation with mortality of subphenotypes of respiratory failure identified with clinical and biological data [20] and with CT qualitative data [9]. This analysis suggests how the process of interaction between medical statistics (LCA) and artificial intelligence (deep learning analyses on automated segmentation on lung CT images) may be a robust interactive ground to build on and strengthen medical evidence [38].

Because of their early hospital admission, our population included patients that were clinically evaluated during low-flow oxygen administration or ambient air. One out of ten of these patients was admitted to ICU. An open question is whether the role of a specific early non-invasive respiratory support or the need of invasive mechanical ventilation may act differently as outcome modifier in the 2 subphenotypes of spontaneously breathing COVID-19 patients. ICU admission was higher in the subphenotype 1, but no difference in ICU mortality was reported between the 2 subphenotypes, suggesting a similar mortality risk when the patients were admitted to the ICU and underwent mechanical ventilation.

Our study has several strengths. First, this is the first study that analyzes a high number of CT studies with a validated machine learning analysis method in spontaneously breathing COVID-19 patients. Second, we emphasized that we built a latent class model in which we add imaging metrics to clinical and laboratory data to provide a characterization of the morphological lung injury patterns of the identified subphenotypes. Third, this is a multicenter clinical trial in which 7 Italian and 1 center from the San Marino Republic obtained clinical and lung imaging data in a specific subpopulation of COVID-19 patients enrolled in the middle of a global worldwide pandemic. Fourth, patients were enrolled in the same pandemic wave, limiting variation linked to genetic SARS-CoV-2 variants change, and potential treatment/preventative measures identification (e.g. steroids, vaccines).

This study has some limitations. First, this is a retrospective observational cohort study of data collected in the middle of a global pandemic, so we could not perform an external validation. However, data were collected from different centers during the first pandemic European wave. Second, we had missing data forcing us to reduce the population size from 810 to 559 patients to build LCA. Consequently, we reported a comprehensive description of differences between the cohort of patients with complete and uncomplete data for LCA. Third, we had limited data on BMI and D-Dimers because of the pandemic surge. However, although in a reduced sample size, BMI did not differ between the subphenotypes, while D-Dimer levels were significantly higher in the subphenotype 1, suggesting a higher proportion of endothelial dysfunction-correlated comorbidities. Furthermore, lung CT data did not include angiograms [35] or CT techniques exploring gas:blood volume mismatch that may serve as proxies of impaired lung perfusion [39, 40]. However, as previously mentioned the higher levels of D-Dimer in the subphenotype 1 may suggest a higher probability of lung malperfusion [35] as compared with the subphenotype 2. Fourth, the biomarkers included in these analyses were limited to those that were measured in an emergency setting but were in line with previous work [5, 8, 31]. Consideration of these biomarkers, and/or of alternative proteomic, genomic or metabolomic markers may recognize these biomarkers as important subphenotye classifiers.

In conclusion, during the first pandemic wave in a western country, we identified two different subphenotypes by LCA on clinical, biological and lung-CT data in COVID-19 patients who were studied while spontaneously breathing and shortly after admission. The subphenotypes were differently associated with hospital mortality and were robust to adjustment for clinically meaningful variables. These findings suggest a potential role of lung imaging in subphenotyping patients with acute respiratory failure, provided that images are objectively analyzed, a task now made possible by machine learning.

### Supplementary Information

Supplementary Material 1.

## Data Availability

The datasets generated and/or analysed during the current study are not publicly available due to local regulations but are available from the corresponding author on reasonable request.

## Abbreviations

ACE: Angiotensin converting enzyme
AIC: Akaike information criterion
ARB: Angiotensin receptor blockers
ARDS: Acute respiratory distress syndrome
BIC: Bayesian information criterion
BMI: Body mass index
CT: Computed tomography
CNN: Convolutional neural network
COPD: Chronic obstructive pulmonary disease
cPAP: Continuous positive airway pressure
ED: Emergency department
GGO: Ground-glass opacities
hs-CRP: High sensitivity C-reactive protein
HU: Hounsfield units
ICU: Intensive care unit
IQR: Interquartile range
LCA: Latent class analysis
LFO: Low-flow oxygen
LMWH: Low molecular weight heparin
LOS: Length of stay
OSAS: Obstructive sleep apnea syndrome
PaCO_2_: Carbon dioxide arterial partial pressure
PaO_2_/FiO_2_: Oxygen arterial partial pressure/inspiratory oxygen fraction
SD: Standard deviation
UFH: Unfractionated heparin
WBC: White blood cells
