# Supplementary material for: Phenotyping COVID-19 respiratory failure in spontaneously breathing patients with AI on lung CT-scan
Source: Crit Care. 2024 Aug 5;28:263. doi: 10.1186/s13054-024-05046-3 (PMC11301830; doi:10.1186/s13054-024-05046-3)
Supplement: Supplementary file 1 — Supplementary Material 1. [file 13054_2024_5046_MOESM1_ESM.docx]

**Supplemental material**

**Phenotyping COVID-19 respiratory failure in spontaneously breathing patients with AI on lung CT-scan.**

Emanuele Rezoagli, MD, PhD^1,2^ Yi Xin, PhD^3,4^ Davide Signori, MD^1^ Wenli Sun, PhD^5^ Sarah Gerard, PhD^6^, Kevin L. Delucchi, PhD^7^ Aurora Magliocca, MD, PhD^8,9^ Giovanni Vitale, MD^8^ Matteo Giacomini, MD^8^ Linda Mussoni, MD^10^ Jonathan Montomoli, MD, PhD^11^ Matteo Subert, MD^12^ Alessandra Ponti, MD^13^ Savino Spadaro, MD^14,15^ Giancarla Poli, MD^16^ Francesco Casola, PhD^17,18^ Jacob Herrmann, PhD^6^ Giuseppe Foti, MD^1,2^ Carolyn S. Calfee, MD^19,20^ John Laffey, MD^21,22^ Giacomo Bellani, MD, PhD^1,2^ Maurizio Cereda, MD^3,4^ for the CT-COVID19 multicenter study group

**Materials and methods**

*Ethical consideration and data acquisition*

The study was performed under the Declaration of Helsinki and in agreement with the Italian good clinical practice recommendations (D.M. Sanità del 15/07/97 e s.m.i.) and with the applied healthcare hospital protocols. No change of current clinical practice or clinical protocols in use were taken in place in the enrolled study population. Considering the retrospective nature of the proposed study, we did not anticipate risks nor benefits that might be added to the patients. Moreover, in the presence of technical difficulties related to the emergency health context to obtain an informed consent from patients in that period of pandemic, informed consent was waived. For this reason and for the great public interest of the project, the research was conducted in the context of the authorizations guaranteed by Article 89 of the GDPR EU Regulation 2016/679, which guarantees the treatment for purposes of public interest, scientific or historical research or for statistical purposes of health data. Personal data were handled in compliance with the European Regulation on the Protection of Personal Data (GDPR), the Legislative Decree 196/2003 and subsequent amendments and additions, and any other Italian law applicable to the protection of personal data (henceforth referred to as the "applicable data protection law"). Data were collected in a pseudo-anonymous way through paper case report forms, digitalized on a University of Milano-Bicocca Institutional Google drive account and analyzed by the scientific coordinator of the project (E.R.). Favorable judgment for the execution of the study was obtained before data acquisition from the local institutional review board of the coordinating center Fondazione IRCCS San Gerardo dei Tintori, Monza, Italy (Approval date: 24/04/2020; number 3375) and from the local institutional review board of each enrolled center (Policlinico San Marco, Gruppo Ospedaliero San Donato, Zingonia, Bergamo, Italy; Ospedale Infermi, Rimini, Italy; Ospedale Papa Giovanni XXIII, Bergamo, Italy; Ospedale Alessandro Manzoni, Lecco, Italy; Arcispedale Sant’Anna, Ferrara, Italy; Ospedale Santa Maria delle Stelle, Melzo, Italy; Istituto Sicureza Sociale, Repubblica di San Marino).

Baseline characteristics (age, sex, body mass index, comorbidities) and clinical illness severity (Sequential Organ Failure Assessment (SOFA) and pH) were collected, together with laboratory biomarkers, blood gas analysis, respiratory assistance, and hemodynamic data at hospital admission. Lung CT scans acquired for clinical purposes within the first week since hospital admission were obtained. Data on drug treatments and complications during hospital admissions, outcomes including length of stay (in ICU and in hospital), use of non-invasive respiratory support, mechanical ventilation-free days, limitation of life sustaining measures, ICU mortality, and hospital mortality were recorded.

*Inclusion and exclusion criteria*

This is a multicenter observational cohort study registered at clinicaltrials.gov as NCT04395482.

Inclusion criteria:

1. patients 18 years old or above;

2. positive confirmation of SARS-CoV-2 infection with nucleic acid amplification test or serology of SARS-CoV2 by nasopharyngeal swab, broncho-aspirate sample or bronchoalveolar lavage;

3. lung CT scan performed within 7 days since hospital admission.

Exclusion criteria:

1. Patients undergoing mechanical ventilation during CT acquisition;
2. Patients with incomplete data to develop the LCA model using clinical, biological and computed tomography data.

For the current analysis we included patients who were admitted to the Emergency Department with a clinical diagnosis of COVID-19 respiratory failure.

*Chest Computed Tomography Quantification*

The lung CT scan images were collected and anonymized by the University of Milano-Bicocca and then transferred via the institutional Google Drive account to the University of Pennsylvania's Department of Anesthesiology and Critical Care and the Department of Radiology (M.C., Y.X., S.G., J.H.) in a de-identified format for advanced quantitative analysis using artificial intelligence and deep learning algorithms [1].

CT images were segmented using an established convolutional neural network (CNN), previously validated in COVID-19 lungs [2]. The masks included vasculature and airways inside the lungs, but excluded major airways (e.g., trachea) and vessels outside the lung lobes in the hilum area. After segmentation, whole-lung and lobar lung masks were inspected and manually adjusted by a trained investigator **(Y.X.)** using ITK-snap software [3].

The whole lung segmentation mask separated different lung regions of interest (ROIs) that were defined by the three axes of space (namely the ventro-dorsal, apical-basal, and submantellar-hilar axes) to explore the potential regional patterns of injury from the gravity gradient in lying down and standing positions, as well as injuries suggested to originate from peripheral lung parenchyma.

The lung lobes of the two different hemithoraxes (i.e., right upper lobe (RUL), right middle lobe (RML), right lower lobe (RLL), left upper lobe (LUL), and left lower lobe (LLL)) were also analyzed separately. Therefore, each lung was divided into fifteen regions-of-interest (ROIs) for subsequent analysis. ROIs included **the following ones:**

- entire **whole** lung;
- five individual lobes (left upper lobe (LUL), left lower lobe (LLL), right upper lobe (RUL), right middle lobe (RML), and right lower lobe (RLL));
- **the analysis by the 3 axes of space (i.e. X,Y and Z) that were three equally sized (by pixel counts) including** **horizontal ventral-to-dorsal regions (Ventral; Dorso-Ventral; Dorsal), vertical apical-to-basal regions (Apical; Basal-Apical; Basal), and three concentric submantellar-to-hilar regions (Submantellar; Central; Hilar~~).~~** three equally sized horizontal ventral-to-dorsal regions, three equal-sized vertical apical-to-basal regions, and three concentric submantellar-to-hilar regions~~.~~ The latter were obtained using an imaging erosion technique with an adjustable erosion radius to achieve three regions with comparable voxel counts [4].

For each ROI, six parameters were analyzed [5,6]:

- average CT density in Hounsfield Units (HU);
- lung gas volume **by density analysis**;
- lung weight by density analysis;
- percentage of consolidated tissue (CT density > -200 HU);
- percentage of ground glass opacity (**-200 HU>CT density>-750 HU**);and
- percentage of total injury [7].

In total, ninety lung features were calculated for each patient, consisting of six parameters for each of fifteen regions. We calculated the gravitational (ventro-dorsal), apical-basal, and submantellar-hilar lung density gradients by linear fitting the density, percentage of GGO, and percentage of consolidation in three corresponding regions. Each set of three numbers was linearly fitted to determine the gradient and compared between latent classes.

Gas volume = CT voxel size * (Mean HU / -1000)

Lung weight = CT voxel size * [1 - (Mean HU / -1000)]

*Latent Class Analysis*

Latent class analysis (LCA) is a well-established statistical technique that employs mixture modeling to identify the most appropriate model for a data set, based on the premise that the data encompasses several unobserved groups or classes. Unlike traditional regression analyses, which aim to delineate the relationship between pre-defined independent variables and a specified outcome, LCA identifies potential subgroups within the data based on combinations of baseline variables, without necessarily linking them to an outcome.

We rigorously implemented LCA following the methodological guidelines to LCA as described by Sinha P. et al. [8], by amalgamating mixed clinical, laboratory, and CT data. Decision on the variables included in the LCA model was based on clinical illness severity at hospital admission and on previously published work [9, 10]. These 15 variables included: Age; Temperature; variables related to gas exchange severity and metabolic status (i.e. PaO_2_/FiO_2_, PCO_2_, and HCO_3_^-^); biomarkers related to inflammation (i.e., WBC, CRP); coagulation activity (i.e., platelets count); variables related to organ functions (i.e. total bilirubin, creatinine); variables related to whole quantitative lung computed tomography data (i.e. mean HU, lung gas volume and lung weight); and variables related to categories of whole lung computed tomography lesions (i.e. proportion of ground glass opacities and consolidation). Full LCA including all the preliminarily selected variables violated model independence constraints. High correlation was explored and the correlation matrix was plotted in **online supplemental Figure 1**. The absolute value of correlations between five pairs (HCO3-, PaCO_2_), (Lung gas volume, GGO), (Lung gas volume, Mean HU), (GGO, Mean HU), (Consolidation, Mean HU) was greater than 0.7, indicating strong correlations [11]. Therefore, mean lung HU, GGO (proportion of ground glass opacities) and HCO_3_^-^ were removed to avoid high correlation. The final 12 variables were PaO_2_/FiO_2_, Lung gas volume, Temperature, PaCO_2_, Total Bilirubin, Platelets, Age, Lung mass, Creatinine, hs-CRP, WBC, Consolidation fraction. We did not include any outcome variables in the modelling.

To address issues with missing data across these variables, we narrowed down our sample size to 559 participants with complete data. While data on CT were available in all patients for inclusion criteria, data about clinical characteristics or laboratory biomarkers were missing across different centers for random reasons, including the paucity of resources during the first COVID-19 pandemic wave and the different clinical admission protocols between different Institutions in which some of the data could be available or not.

All the aforementioned 12 variables from 559 samples were included in the LCA model with different numbers of classes and different specifications of covariance matrix structures. Depending on the model configuration, the identified classes can show different class-specific covariances [12]. We explored three settings of covariance-variance structure as shown in **online supplemental Figure 2**. The freely-estimated sample residual variance and covariances model (blue line) fit best in terms of smallest Bayesian Information Criterion (BIC). Under the assumption of freed variance and covariances, we compare the BIC and the averaged uncertainty across entire samples among 2, 3, 4, 5 and 6 classes. The optimal model that yielded the smallest BIC (30654) and uncertainty (2.8%) was the one with two-classes. In addition, entropy was computed as a measure of effective separation between the latent classes. However, it is not a reliable sole criterion for choosing the best model because a model that overfits may also exhibit high entropy; therefore entropy should be evaluated in the context of the smallest BIC and uncertainty [8]. The BIC, averaged uncertainty and entropy measures for each model are listed in **Supplemental Table 1**. As a result, we retained the two-class model: Class 1 (N=156) and Class 2 (N=403).

*Statistical analysis*

Continuous data are reported as mean ± standard deviation (SD) or median and interquartile range (IQR) according to data distribution evaluated by the Shapiro-Wilk test and by visual inspection. Categorical variables are expressed as proportions (frequency). Differences between the 2 clusters were assessed by unpaired Student’s T-test or U Mann-Whitney test as appropriate. Differences between categorical data were assessed by using Pearson’s chi-square test or Fisher’s exact test. Correlation between quantitative lung computed tomography data and gas exchange was assessed by linear regression analysis and Pearson correlation coefficient was reported. Differences in 90-day survival across subphenotypes was explored by Kaplan-Meier approach and reported by log-rank p-value. Patients who were discharged alive from the hospital before 90-day follow-up were considered alive at 90 days. To assess association between subphenotypes and 90-day mortality, univariable Cox proportional regression was tested. To explore the independent association of subphenotypes with 90-day mortality, multivariable Cox proportional regression models were tested and Hazard ratios with 95% confidence interval were estimated. Clinically meaningful covariates were decided a priori to adjust the multivariable models as follows: sex, the presence of any comorbidities, the decision of limitation of life sustaining measures. Adjusted models were ranked by their Akaike information criterion (AIC) and their BIC. AIC and BIC address both goodness-of-fit and simplicity of a model. Since we compared models with the same number of independent variables for the same set of patients, the lowest AIC and BIC represented the best fit model. Further, we investigated LCA modeling by only including clinical and laboratory data (i.e. PaO_2_/FiO_2_, Temperature, PaCO_2_, Total Bilirubin, Platelets, Age, Creatinine, hs-CRP, WBC) or only including CT derived features (i.e. Lung gas volume, Lung mass, Consolidation fraction) to assess whether the most complete LCA model including overall mixed clinical, laboratory, and CT data showed a better association with 90-day mortality and the highest goodness of model fitting.

Statistical significance was considered with a p<0.05 (two-tailed). Statistical analysis was performed by SPSS software v28 (IBM Corp., Armonk, NY, USA), R-project (Version 4.3.2) and Stata/MP 17.0 (Copyright 1985-2021 StataCorp LLC (College Station, TX, 77845, USA).

*Sample size*

We aimed to collect data from 500 patients at least, as this is considered an adequate sample size to conduct LCA and to detect the correct number of latent classes [8].

Comprehensive information on methods is reported in the Supplemental material.

The Strengthening the Reporting of Observational studies in Epidemiology (STROBE) reporting guideline checklist for observational studies was used for reporting this study.

**References**

1. Transfer Learning for Segmentation of Injured Lungs Using Coarse-to-Fine Convolutional Neural Networks - University of Iowa. https://iro.uiowa.edu/esploro/outputs/conferenceProceeding/Transfer-Learning-for-Segmentation-of-Injured/9984006440502771. Accessed 14 May 2023

2. Gerard SE, Herrmann J, Xin Y, Martin KT, Rezoagli E, Ippolito D, Bellani G, Cereda M, Guo J, Hoffman EA, Kaczka DW, Reinhardt JM (2021) CT image segmentation for inflamed and fibrotic lungs using a multi-resolution convolutional neural network. *Sci Rep.* 11:1455.

3. Yushkevich PA, Piven J, Hazlett HC, Smith RG, Ho S, Gee JC, Gerig G (2006) User-guided 3D active contour segmentation of anatomical structures: significantly improved efficiency and reliability. *Neuroimage*. 31:1116–1128.

4. Pellegrini M, Larina A, Mourtos E, Frithiof R, Lipcsey M, Hultström M, Segelsjö M, Hansen T, Perchiazzi G (2021) A quantitative analysis of extension and distribution of lung injury in COVID-19: a prospective study based on chest computed tomography. *Crit Care*. 25:276.

5. Gattinoni L, Pesenti A, Avalli L, Rossi F, Bombino M (1987) Pressure-volume curve of total respiratory system in acute respiratory failure. Computed tomographic scan study. *Am Rev Respir Dis.* 136:730–736.

6. Kauczor HU, Heitmann K, Heussel CP, Marwede D, Uthmann T, Thelen M (2000) Automatic detection and quantification of ground-glass opacities on high-resolution CT using multiple neural networks: comparison with a density mask. *AJR Am J Roentgenol*. 175:1329–1334.

7. Mortani Barbosa EJ, Georgescu B, Chaganti S, Aleman GB, Cabrero JB, Chabin G, Flohr T, Grenier P, Grbic S, Gupta N, Mellot F, Nicolaou S, Re T, Sanelli P, Sauter AW, Yoo Y, Ziebandt V, Comaniciu D (2021) Machine learning automatically detects COVID-19 using chest CTs in a large multicenter cohort. *Eur Radiol*. 31:8775–8785.

8. Sinha P, Calfee CS, Delucchi KL (2021) Practitioner’s Guide to Latent Class Analysis: Methodological Considerations and Common Pitfalls. *Crit Care Med.* 49:e63–e79.

9. Calfee CS, Delucchi KL, Sinha P, Matthay MA, Hackett J, Shankar-Hari M, McDowell C, Laffey JG, O’Kane CM, McAuley DF, Irish Critical Care Trials Group (2018) Acute respiratory distress syndrome subphenotypes and differential response to simvastatin: secondary analysis of a randomised controlled trial. *Lancet Respir Med*. 6:691–698.

10. Calfee CS, Delucchi K, Parsons PE, Thompson BT, Ware LB, Matthay MA, NHLBI ARDS Network (2014) Subphenotypes in acute respiratory distress syndrome: latent class analysis of data from two randomised controlled trials. L*ancet Respir Med*. 2:611–620.

11. Ratner B (2009) The correlation coefficient: Its values range between +1/−1, or do they? *J Target Meas Anal Mark*. 17:139–142.

12. Celeux G, Govaert G (1995) Gaussian parsimonious clustering models. *Pattern Recognition.* 28:781–793.

**Supplemental Tables**

**Supplemental Table 1. BIC, averaged uncertainty, and entropy measures across entire samples among 1, 2, 3, 4, 5 and 6 classes.**

| **Latent Classes** | **1**  Class 1, n=559 | **2**  Class 1, n=156  Class 2, n=403 | **3**  Class 1, n=223  Class 2, n=27  Class 3, n=309 | **4**  Class 1, n=184  Class 2, n=25  Class 3, n=215  Class 4, n=135 | **5**  Class 1, n=122  Class 2, n=24  Class 3, n=188  Class 4, n=113  Class 5, n=112 | **6**  Class 1, n=100  Class 2, n=12  Class 3, n=144  Class 4, n=143  Class 5, n=117  Class 6, n=43 |
| --- | --- | --- | --- | --- | --- | --- |
| **Least BIC** | 32091 | 30654 | 30587 | 30871 | 31198 | 31614 |
| **Average Uncertainty** | NA | 2.8% | 5.7% | 9.2% | 10.8% | 9.6% |
| **Entropy** | NA | 0.89 | 0.89 | 0.88 | 0.89 | 0.91 |

**Supplemental Table 2.** Continuous variables differences by LCA derived subphenotype expressed as subphenoype 1 and 2.

| **LCA variables** | **Overall (N=559)** | **Subphenotype 1 (N=156)** | **Subphenotype 2**  **(N=403)** | **Mean difference**  **(95% CI)** | **p-value** |
| --- | --- | --- | --- | --- | --- |
| **Clinical Variables** |  |  |  |  |  |
| Temperature (°C) | 37.7 (1) | 37.6 (1) | 37.7 (1) | -0.1 [-0.3;0] | 0.135 |
| Age (years) | 67 (14) | 73 (13) | 65 (14) | 8 [6;11] | <0.001 |
| **Laboratory variables** |  |  |  |  |  |
| PaO_2_/FiO_2_ (mmHg) | 252 (103) | 188 (95) | 277 (95) | -89 [-107;-72] | <0.001 |
| PaCO_2_ (mmHg) | 33 (6) | 33 (7) | 33 (5) | 1 [0;2] | 0.221 |
| Total bilirubin (mg/dl) | 0.6 (0.4) | 0.7 (0.6) | 0.6 (0.3) | 0.1 [0;0.2] | 0.025 |
| Platelets (x1000/µL) | 214 (95) | 252 (119) | 199 (80) | 53 [36;70] | <0.001 |
| Creatinine (mg/dl) | 1.3 (1) | 2 (1.6) | 1.1 (0.3) | 0.9 [0.7;1.1] | <0.001 |
| CRP (mg/dl) | 10.5 (9.5) | 16.4 (13.2) | 8.1 (6.2) | 8.3 [6.7;9.9] | <0.001 |
| WBC (x1000/μL) | 7.5 (3.72) | 10.29 (4.91) | 6.42 (2.39) | 3.87 [3.26;4.48] | <0.001 |
| **CT derived features** |  |  |  |  |  |
| Lung gas volume (l) | 2.91 (1.25) | 2.16 (0.9) | 3.20 (1.25) | -1.04 [-1.26;-0.83] | <0.001 |
| Lung weight (Kg) | 1.1 (0.3) | 1.28 (0.42) | 1.05 (0.27) | 0.23 [0.17;0.29] | <0.001 |
| Consolidation (fraction) | 0.07 (0.06) | 0.12 (0.09) | 0.05 (0.03) | 0.07 [0.06;0.08] | <0.001 |

Variables were grouped as clinical and laboratory variables and computed tomography derived features. In each group, variables are sorted based on the degree of separation between the subphenotypes, from maximum positive separation (ie, subphenotype 2 higher than subphenotype 1) to maximum negative separation (ie, subphenotype 2 lower than subphenotype 1). The y-axis describes the standardized variable values, in which all means are scaled to zero and standard deviations (SDs) to one. Differences between the 2 subphenotypes of LCA explored variables were assessed and reported as mean difference (95% CI) and p-value. Data are expressed as mean (standard deviation). CRP: C-reactive protein; CT: computed tomography; PaCO_2_: arterial carbon dioxide partial pressure; PaO_2_/FiO_2_: ratio of arterial oxygen partial pressure to inspiratory oxygen fraction; WBC: white blood cells. CT data are referred to whole lungs.

**Supplemental Table 3. Baseline characteristics, comorbidities, clinical illness severity, respiratory support at hospital admission, treatments, and outcomes of patients stratified by LCA analysis inclusion.**

|  | | | **N** | **Overall** | **LCA**  **Included** | **LCA**  **Excluded** | **p-value** |
| --- | --- | --- | --- | --- | --- | --- | --- |
| **Reason of hospital admission** | | |  |  |  |  |  |
| Respiratory failure, n (%) | | | 810 | 810 | 559 | 251 | - |
| **Baseline characteristics** | | |  |  |  |  |  |
| BMI, kg/m^2^, mean (standard deviation) | | | 328 | 27.9 (4.8) | 27.7 (4.6) | 28.5 (5.4) | 0.261 |
| Sex, F (%) | | | 810 | 287 (35) | 196 (35) | 91 (36) | 0.743 |
| Time between admission and CT scan, days, median (IQR) | | | 786 | 0 [0;0] | 0 [0;0] | 0 [0;1] | <0.001 |
| **Comorbidities** | | |  |  |  |  |  |
| COPD, n (%) | | | 810 | 46 (5.7) | 28 (5) | 18 (7.2) | 0.219 |
| Asthma, n (%) | | | 810 | 35 (4.3) | 27 (4.8) | 8 (3.2) | 0.288 |
| Congestive heart failure, n (%) | | | 810 | 35 (4.3) | 26 (4.7) | 9 (3.6) | 0.490 |
| Chronic kidney disease, n (%) | | | 810 | 50 (6.2) | 37 (6.6) | 13 (5.2) | 0.431 |
| Chronic liver failure, n (%) | | | 810 | 6 (0.7) | 3 (0.5) | 3 (1.2) | 0.312 |
| Solid cancer, n (%) | | | 810 | 29 (3.6) | 14 (2.5) | 15 (6) | 0.014 |
| Hematologic malignancy, n (%) | | | 810 | 13 (1.6) | 9 (1.6) | 4 (1.6) | 0.986 |
| Immune mediated disease, n (%) | | | 810 | 31 (3.8) | 20 (3.6) | 11 (4.4) | 0.581 |
| Diabetes, n (%) | | | 810 | 135 (16.7) | 97 (17.4) | 38 (15.1) | 0.434 |
| Systemic hypertension, n (%) | | | 810 | 399 (49.3) | 280 (50.1) | 119 (47.4) | 0.481 |
| Antihypertensive drugs, n (%) | | | 779 |  |  |  | 0.040 |
| - ACE-inhibitors | | |  | 503 (64.6) | 346 (63) | 157 (68.3) |  |
| - ARBs | | |  | 131 (16.8) | 90 (16.4) | 41 (17.8) |  |
| - Others/Unknown | | |  | 145 (18.6) | 113 (20.6) | 32 (13.9) |  |
| Pulmonary hypertension, n (%) | | | 810 | 4 (0.5) | 3 (0.5) | 1 (0.4) | 0.795 |
| Atrial fibrillation, n (%) | | | 810 | 77 (9.5) | 57 (10.2) | 20 (8) | 0.317 |
| OSAS, n (%) | | | 809 | 13 (1.6) | 6 (1.1) | 7 (2.8) | 0.071 |
| cPAP at home, n (%) | | | 810 | 8 (1) | 4 (0.7) | 4 (1.6) | 0.243 |
| Pacing, n (%) | | | 810 | 13 (1.6) | 10 (1.8) | 3 (1.2) | 0.534 |
| Any comorbidities, n (%) | | | 810 | 556 (69) | 404 (72) | 152 (61) | <0.001 |
| **Clinical illness severity** | | |  |  |  |  |  |
| pH, mean (standard deviation) | | | 704 | 7.46 (0.05) | 7.47 (0.05) | 7.46 (0.05) | 0.422 |
| FiO_2_, median (IQR) | | | 784 | 21 [21;60] | 21 [21;60] | 29 [21;50] | 0.075 |
| Dimers, mean (standard deviation), n=287 | | | 357 | 3.17 (8.49) | 2.72 (5.93) | 5 (14.89) | 0.044 |
| **Respiratory support** | | | 790 |  |  |  |  |
| Oxygen delivery, n (%) | | Room air |  | 523 (66.2) | 367 (65.8) | 156 (67.2) | <0.001 |
|  |  | LFO |  | 250 (31.6) | 189 (33.9) | 61 (26.3) |  |
| Non-invasive ventilation, n (%) | | cPAP |  | 17 (2.2) | 2 (0.4) | 15 (6.5) |  |
| **Pharmacological treatments** | | |  |  |  |  |  |
| Antivirals, n (%) | | | 797 | 350 (43.9) | 202 (36.3) | 148 (61.7) | <0.001 |
| Antibiotic treatment, n (%) | | | 796 | 658 (82.7) | 491 (88.3) | 167 (69.6) | <0.001 |
| Steroids, n (%) | | | 793 | 205 (25.9) | 128 (23.1) | 77 (32.1) | 0.008 |
| Hydroxycloroquine, n (%) | | | 796 | 575 (72.2) | 406 (73.2) | 169 (70.1) | 0.381 |
| Cloroquine phosphate, n (%) | | | 795 | 81 (10.2) | 48 (8.7) | 33 (13.7) | 0.031 |
| Immunoglobulins IV, n (%) | | | 794 | 2 (0.3) | 2 (0.4) | 0 (0) | 0.35 |
| Tocilizumab, n (%) | | | 793 | 59 (7.4) | 30 (5.4) | 29 (12.1) | 0.001 |
| Anakinra, n (%) | | | 789 | 15 (1.9) | 4 (0.7) | 11 (4.6) | <0.001 |
| Thrombolysis, n (%) | | | 792 | 3 (0.4) | 2 (0.4) | 1 (0.4) | 0.905 |
| Anticoagulation, n (%) | | | 792 | 521 (65.8) | 379 (68.7) | 142 (59.2) | 0.01 |
| Anticoagulant | None, n (%) | | 782 | 223 (28.5) | 149 (27.1) | 74 (31.9) | 0.110 |
|  | LMWH, n (%) | |  | 511 (65.3) | 371 (67.5) | 140 (60.3) |  |
|  | UFH, n (%) | |  | 7 (0.9) | 2 (0.4) | 5 (2.2) |  |
|  | Argatroban, n (%) | |  | 22 (2.8) | 16 (2.9) | 6 (2.6) |  |
|  | Fondaparinux, n (%) | |  | 3 (0.4) | 2 (0.4) | 1 (0.4) |  |
|  | Salicilic acid, n (%) | |  | 16 (2) | 10 (1.8) | 6 (2.6) |  |
| **Organ support techniques** | | |  |  |  |  |  |
| CRRT, n (%) | | | 793 | 17 (2.1) | 9 (1.6) | 8 (3.3) | 0.128 |
| **Complications** | | |  |  |  |  |  |
| All Bacterial overinfections, n (%) | | | 790 | 98 (12.4) | 57 (10.3) | 41 (17.5) | 0.005 |
| Lung overinfection, n (%) | | | 790 | 49 (6.2) | 33 (5.9) | 16 (6.8) | 0.631 |
| Blood overinfection, n (%) | | | 788 | 25 (3.2) | 17 (3.1) | 8 (3.4) | 0.787 |
| Urinary tract overinfection, n (%) | | | 789 | 17 (2.2) | 9 (1.6) | 8 (3.4) | 0.112 |
| Soft tissues overinfection, n (%) | | | 789 | 4 (0.5) | 4 (0.7) | 0 (0) | 0.193 |
| Abdominal overinfection, n (%) | | | 789 | 3 (0.4) | 2 (0.4) | 1 (0.4) | 0.889 |
| Stroke, n (%) | | | 791 | 9 (1.1) | 6 (1.1) | 3 (1.3) | 0.838 |
| Venous thromboembolism, n (%) | | | 791 | 15 (1.9) | 7 (1.3) | 8 (3.3) | 0.049 |
| Pulmonary thromboembolism, n (%) | | | 788 | 18 (2.3) | 9 (1.6) | 9 (3.8) | 0.06 |
| Tracheostomy, n (%) | | | 810 | 29 (3.6) | 27 (4.8) | 2 (0.8) | 0.04 |
| **Outcomes** | | |  |  |  |  |  |
| MV duration (days), mean (standard deviation) | | | 85 | 15 (14) | 14 (12) | 18 (17) | 0.245 |
| Limitation of life sustaining measures, n (%) | | | 719 | 163 (22.7) | 132 (24.2) | 31 (17.9) | 0.087 |
| ICU admission, n (%) | | | 810 | 97 (12) | 59 (10.6) | 38 (15.1) | 0.063 |
| ICU mortality, n (%) | | | 99 | 49 (49.5) | 35 (57.4) | 14 (36.8) | 0.047 |
| ICU LOS, mean (standard deviation) | | | 94 | 20 (18) | 17 (14) | 24 (23) | 0.05 |
| - Survivors, n (%) | | | 47 | 27 (22) | 22 (16) | 32 (25) | 0.117 |
| - Dead, n (%) | | | 47 | 13 (9) | 14 (10) | 11 (6) | 0.348 |
| Hospital mortality, n (%) | | | 809 | 234 (28.9) | 180 (32.2) | 54 (21.6) | 0.002 |
| Hospital LOS, mean (standard deviation) | | | 802 | 15 (18) | 13 (15) | 18 (23) | 0.001 |
| - Survivors, n (%) | | | 568 | 16 (20) | 14 (16) | 19 (26) | 0.006 |
| - Dead, n (%) | | | 233 | 10 (9) | 11 (10) | 10 (7) | 0.859 |

ACE: angiotensin converting enxyme; ARB: angiotensin receptor blockers; BMI: body mass index; COPD: chronic obstructive pulmonary disease; cPAP: continuous positive airway pressure; CRRT: continuous renal replacement therapy; FiO_2_: inspiratory oxygen fraction; ICU: intensive care unit; LFO: low-flow oxygen; LMWH: low molecular weight heparin; LOS: length of stay; MV: mechanical ventilation; OSAS: obstructive sleep apnea syndrome; UFH: Unfractionated heparin; VV-ECMO: venous-venous extracorporeal membrane oxygenation; Sample size was reported to evaluate the presence of any missing data. Differences between the 2 cohorts were assessed and reported in p-value column. Continuous data are expressed as mean (standard deviation), categorical variables as count (relative frequency %).

| **LCA variables** | **N** | **Overall (n=810)** | **LCA**  **Included (n=559)** | **LCA**  **Excluded (n=251)** | **Mean difference**  **(95% CI)** | **p-value** |
| --- | --- | --- | --- | --- | --- | --- |
| PaO_2_/FiO_2_ (mmHg) | 710 | 254 (106) | 252 (103) | 259 (116) | -6.9 [-27.3;13.6] | 0.51 |
| Lung volume (l) | 810 | 2.97 (1.27) | 2.91 (1.25) | 3.1 (1.32) | -0.2 [-0.4;0] | 0.064 |
| Temperature (°C) | 787 | 37.6 (1) | 37.7 (1) | 37.3 (1) | 0.4 [0.2;0.5] | <0.001 |
| PaCO_2_ (mmHg) | 709 | 33 (6) | 33 (6) | 33 (6) | 0.2 [-0.9;1.3] | 0.704 |
| Total bilirubin (mg/dl) | 672 | 0.63 (0.39) | 0.64 (0.39) | 0.61 (0.37) | 0 [0;0.1] | 0.425 |
| Platelets (x1000/µL) | 791 | 207 (93) | 214 (95) | 193 (84) | 21.1 [7;35.2] | 0.004 |
| Age (years) | 810 | 67 (15) | 67 (14) | 66 (16) | 1 [-1.3;3.3] | 0.393 |
| Lung mass (Kg) | 810 | 1.11 (0.35) | 1.12 (0.33) | 1.09 (0.37) | 0 [0;0.1] | 0.272 |
| Creatinine (mg/dl) | 786 | 1.31 (1.04) | 1.34 (0.96) | 1.24 (1.23) | 0.1 [-0.1;0.3] | 0.313 |
| CRP (mg/dl) | 771 | 9.88 (9.7) | 10.45 (9.5) | 8.39 (10.06) | 2.1 [0.5;3.6] | 0.010 |
| WBC (x1000/μL) | 791 | 7.37 (3.75) | 7.5 (3.72) | 7.06 (3.81) | 0.4 [-0.1;1] | 0.137 |
| Consolidation fraction | 810 | 0.07 (0.06) | 0.07 (0.06) | 0.07 (0.07) | 0.001 [-0.009;0.011] | 0.896 |

**Supplemental Table 4.** Differences in continuous variables used to build the LCA model between cohorts of patients included and excluded in the LCA model.

Data are expressed as mean (standard deviation). WBC: white blood cells; CRP: C-reactive protein; PaCO_2_: arterial carbon dioxide partial pressure; PaO_2_/FiO_2_: ratio of arterial oxygen partial pressure to fractional inspired oxygen. CT data refer to whole lung.

**Supplemental Table 5.** Quantitative and qualitative analysis of lung CT images stratified by cohorts of patients included and excluded in the LCA model.

|  | **Overall (n=810)** | **LCA**  **Included**  **(n=559)** | **LCA**  **Excluded (n=251)** | **p-value** |
| --- | --- | --- | --- | --- |
| **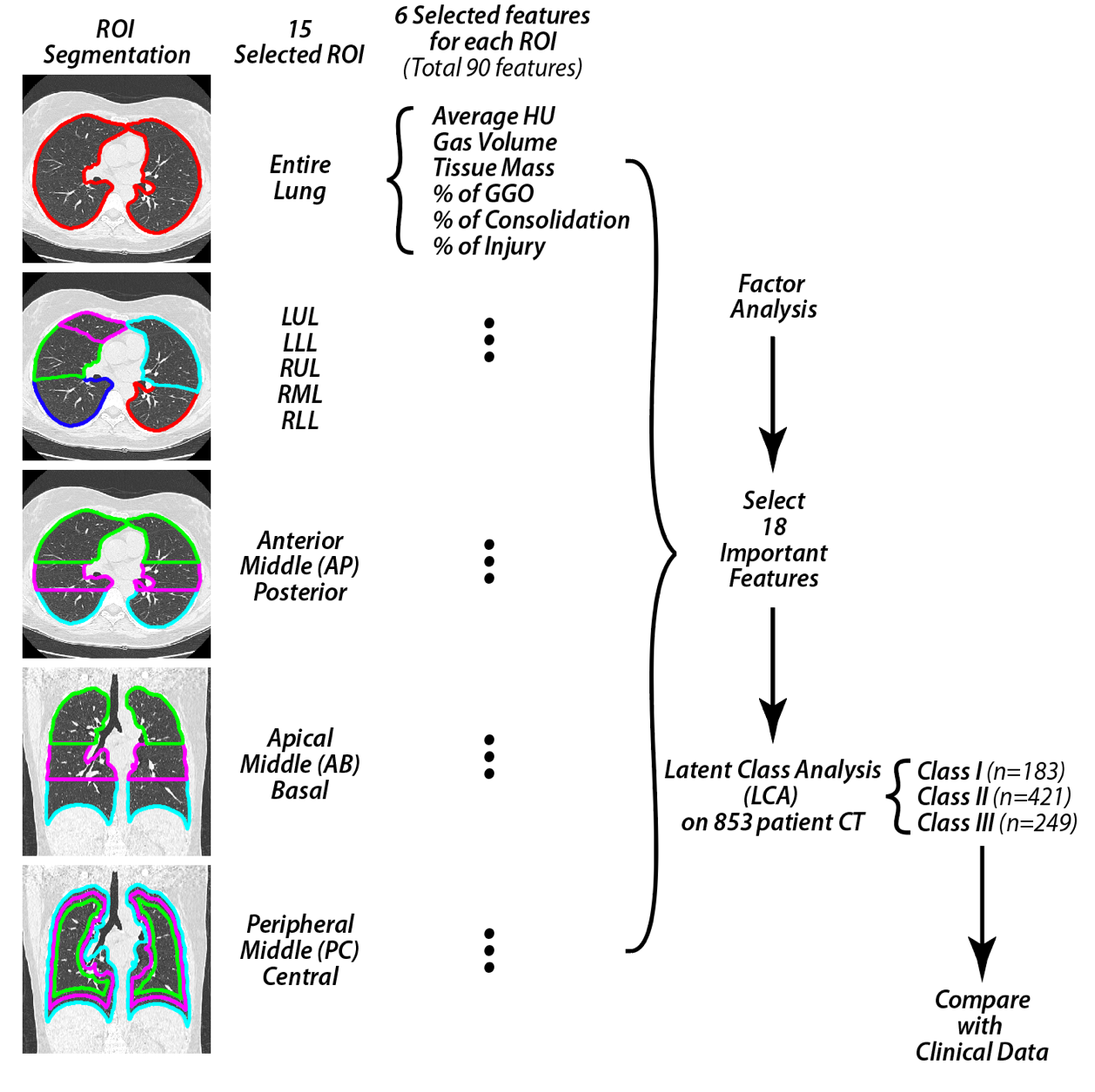** |  |  |  |  |
| **Whole lung** |  |  |  |  |
| Mean lung density (HU) | -707 (108) | -702 (106) | -719 (112) | 0.043 |
| Lung gas volume (L) | 2.97 (1.27) | 2.91 (1.25) | 3.1 (1.32) | 0.064 |
| Lung weight (kg) | 1.11 (0.35) | 1.12 (0.33) | 1.09 (0.37) | 0.272 |
| Total injury (fraction) | 0.41 (0.2) | 0.43 (0.19) | 0.38 (0.2) | 0.002 |
| GGO (fraction) | 0.35 (0.16) | 0.36 (0.16) | 0.32 (0.15) | <0.001 |
| Consolidation (fraction) | 0.07 (0.06) | 0.07 (0.06) | 0.07 (0.07) | 0.896 |
| **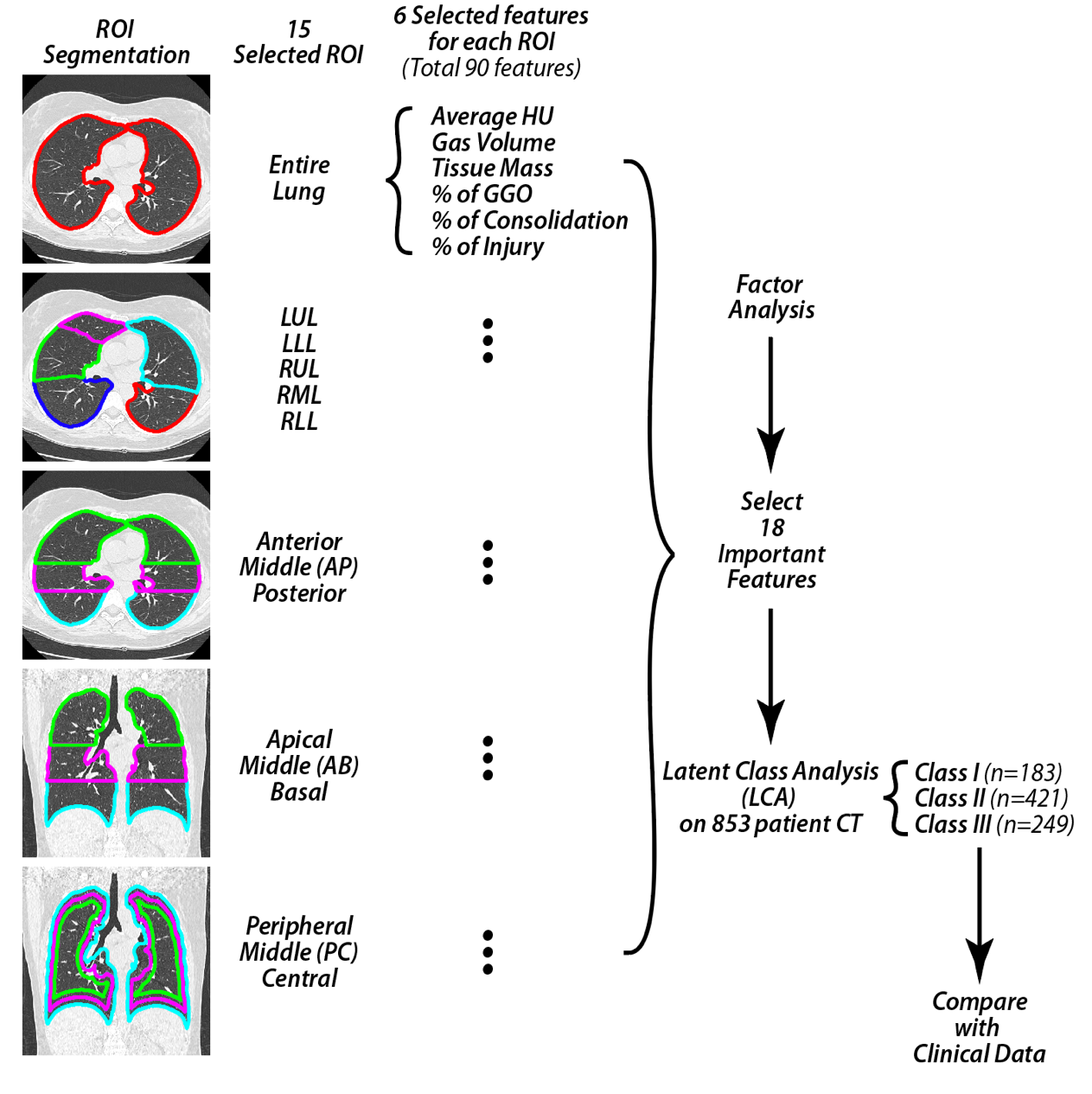** |  |  |  |  |
| **Left Upper Lobe** |  |  |  |  |
| Mean lung density (HU) | -739 (108) | -734 (110) | -749 (103) | 0.057 |
| Lung gas volume (L) | 0.81 (0.35) | 0.8 (0.36) | 0.83 (0.34) | 0.262 |
| Lung weight (kg) | 0.26 (0.09) | 0.26 (0.09) | 0.25 (0.1) | 0.274 |
| Total injury (fraction) | 0.36 (0.2) | 0.38 (0.2) | 0.34 (0.2) | 0.013 |
| GGO (fraction) | 0.32 (0.17) | 0.33 (0.17) | 0.29 (0.17) | 0.006 |
| Consolidation (fraction) | 0.05 (0.06) | 0.05 (0.07) | 0.05 (0.05) | 0.478 |
| **Left Lower Lobe** |  |  |  |  |
| Mean lung density (HU) | -652 (149) | -647 (145) | -665 (155) | 0.124 |
| Lung gas volume (L) | 0.57 (0.33) | 0.55 (0.32) | 0.59 (0.35) | 0.126 |
| Lung weight (kg) | 0.25 (0.1) | 0.26 (0.09) | 0.25 (0.1) | 0.299 |
| Total injury (fraction) | 0.5 (0.23) | 0.52 (0.23) | 0.47 (0.24) | 0.004 |
| GGO (fraction) | 0.4 (0.18) | 0.42 (0.18) | 0.37 (0.17) | <0.001 |
| Consolidation (fraction) | 0.1 (0.12) | 0.1 (0.11) | 0.1 (0.12) | 0.949 |
| **Right Upper Lobe** |  |  |  |  |
| Mean lung density (HU) | -729 (114) | -723 (113) | -741 (115) | 0.037 |
| Lung gas volume (L) | 0.65 (0.29) | 0.64 (0.29) | 0.68 (0.29) | 0.093 |
| Lung weight (kg) | 0.22 (0.08) | 0.22 (0.08) | 0.21 (0.08) | 0.276 |
| Total injury (fraction) | 0.38 (0.21) | 0.39 (0.21) | 0.35 (0.21) | 0.004 |
| GGO (fraction) | 0.32 (0.18) | 0.34 (0.18) | 0.3 (0.17) | 0.001 |
| Consolidation (fraction) | 0.05 (0.07) | 0.06 (0.06) | 0.05 (0.07) | 0.478 |
| **Right Medium Lobe** |  |  |  |  |
| Mean lung density (HU) | -761 (94) | -758 (92) | -768 (99) | 0.156 |
| Lung gas volume (L) | 0.33 (0.15) | 0.32 (0.15) | 0.34 (0.16) | 0.085 |
| Lung weight (kg) | 0.09 (0.05) | 0.09 (0.03) | 0.1 (0.08) | 0.371 |
| Total injury (fraction) | 0.33 (0.19) | 0.34 (0.19) | 0.3 (0.19) | 0.009 |
| GGO (fraction) | 0.29 (0.17) | 0.3 (0.17) | 0.26 (0.16) | <0.001 |
| Consolidation (fraction) | 0.04 (0.04) | 0.04 (0.03) | 0.04 (0.06) | 0.104 |
| **Right Lower Lobe** |  |  |  |  |
| Mean lung density (HU) | -643 (150) | -635 (146) | -659 (156) | 0.044 |
| Lung gas volume (L) | 0.61 (0.35) | 0.6 (0.34) | 0.65 (0.38) | 0.033 |
| Lung weight (kg) | 0.29 (0.11) | 0.29 (0.11) | 0.28 (0.11) | 0.156 |
| Total injury (fraction) | 0.51 (0.24) | 0.53 (0.23) | 0.47 (0.24) | <0.001 |
| GGO (fraction) | 0.41 (0.18) | 0.42 (0.18) | 0.37 (0.17) | <0.001 |
| Consolidation (fraction) | 0.11 (0.11) | 0.11 (0.11) | 0.1 (0.12) | 0.685 |
| **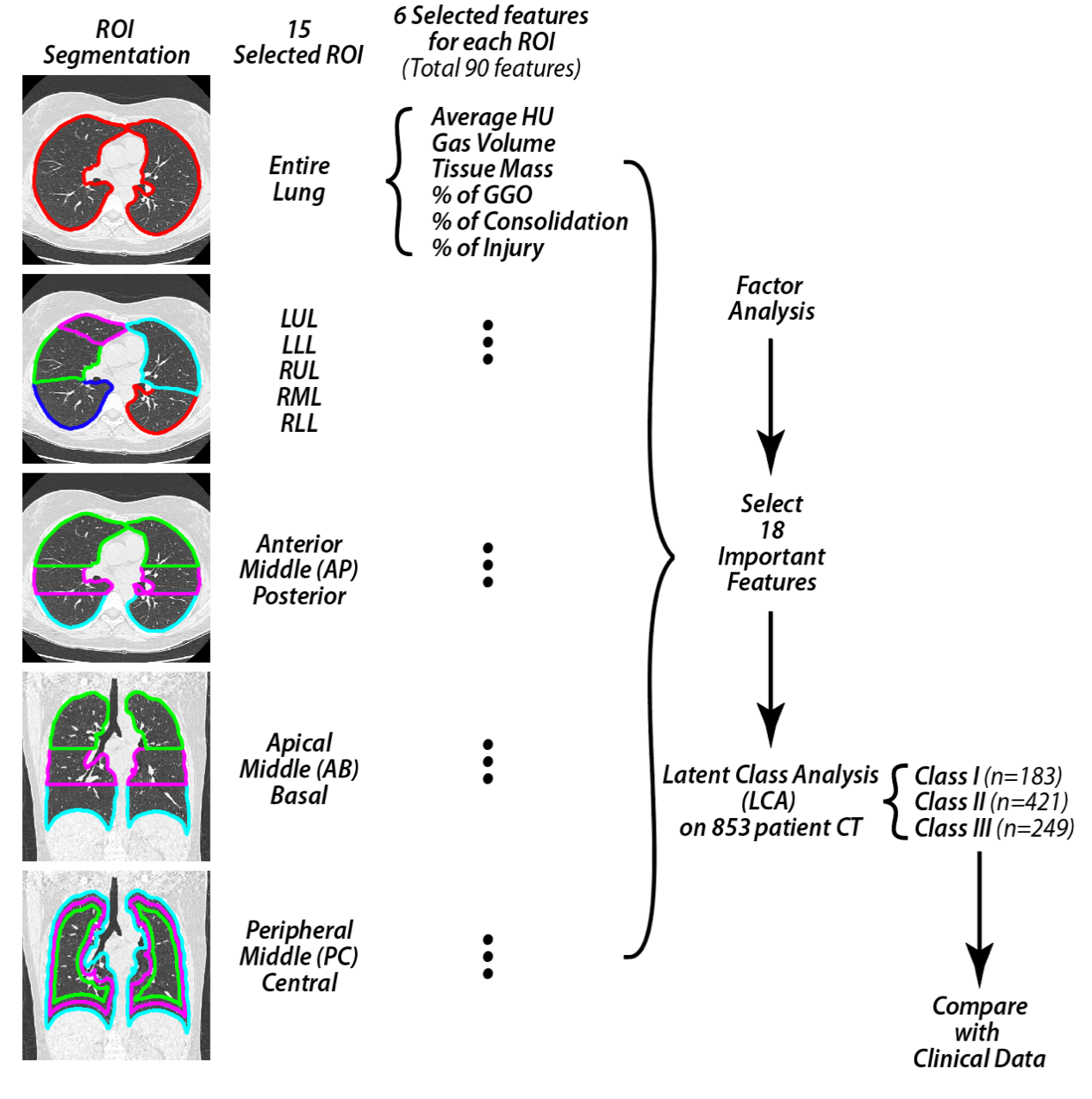** |  |  |  |  |
| **Basal** |  |  |  |  |
| Mean lung density (HU) | -684 (123) | -679 (121) | -696 (127) | 0.068 |
| Lung gas volume (L) | 0.95 (0.43) | 0.93 (0.43) | 0.99 (0.45) | 0.07 |
| Lung weight (kg) | 0.39 (0.13) | 0.39 (0.12) | 0.38 (0.14) | 0.400 |
| Total injury (fraction) | 0.46 (0.22) | 0.47 (0.21) | 0.42 (0.21) | 0.003 |
| GGO (fraction) | 0.38 (0.17) | 0.4 (0.17) | 0.35 (0.16) | <0.001 |
| Consolidation (fraction) | 0.08 (0.08) | 0.08 (0.07) | 0.08 (0.09) | 0.923 |
| **Basal-Apical** |  |  |  |  |
| Mean lung density (HU) | -700 (112) | -695 (109) | -712 (118) | 0.047 |
| Lung gas volume (L) | 0.98 (0.42) | 0.96 (0.41) | 1.02 (0.44) | 0.052 |
| Lung weight (kg) | 0.38 (0.12) | 0.38 (0.12) | 0.37 (0.13) | 0.242 |
| Total injury (fraction) | 0.42 (0.2) | 0.44 (0.2) | 0.39 (0.2) | 0.001 |
| GGO (fraction) | 0.35 (0.16) | 0.36 (0.16) | 0.31 (0.16) | <0.001 |
| Consolidation (fraction) | 0.07 (0.07) | 0.07 (0.06) | 0.07 (0.08) | 0.908 |
| **Apical** |  |  |  |  |
| Mean lung density (HU) | -737 (106) | -732 (106) | -748 (106) | 0.042 |
| Lung gas volume (L) | 1.04 (0.43) | 1.02 (0.42) | 1.08 (0.44) | 0.073 |
| Lung weight (kg) | 0.34 (0.12) | 0.34 (0.12) | 0.33 (0.12) | 0.232 |
| Total injury (fraction) | 0.36 (0.2) | 0.38 (0.2) | 0.34 (0.2) | 0.006 |
| GGO (fraction) | 0.31 (0.16) | 0.33 (0.16) | 0.29 (0.16) | 0.002 |
| Consolidation (fraction) | 0.05 (0.06) | 0.05 (0.06) | 0.05 (0.06) | 0.479 |
| **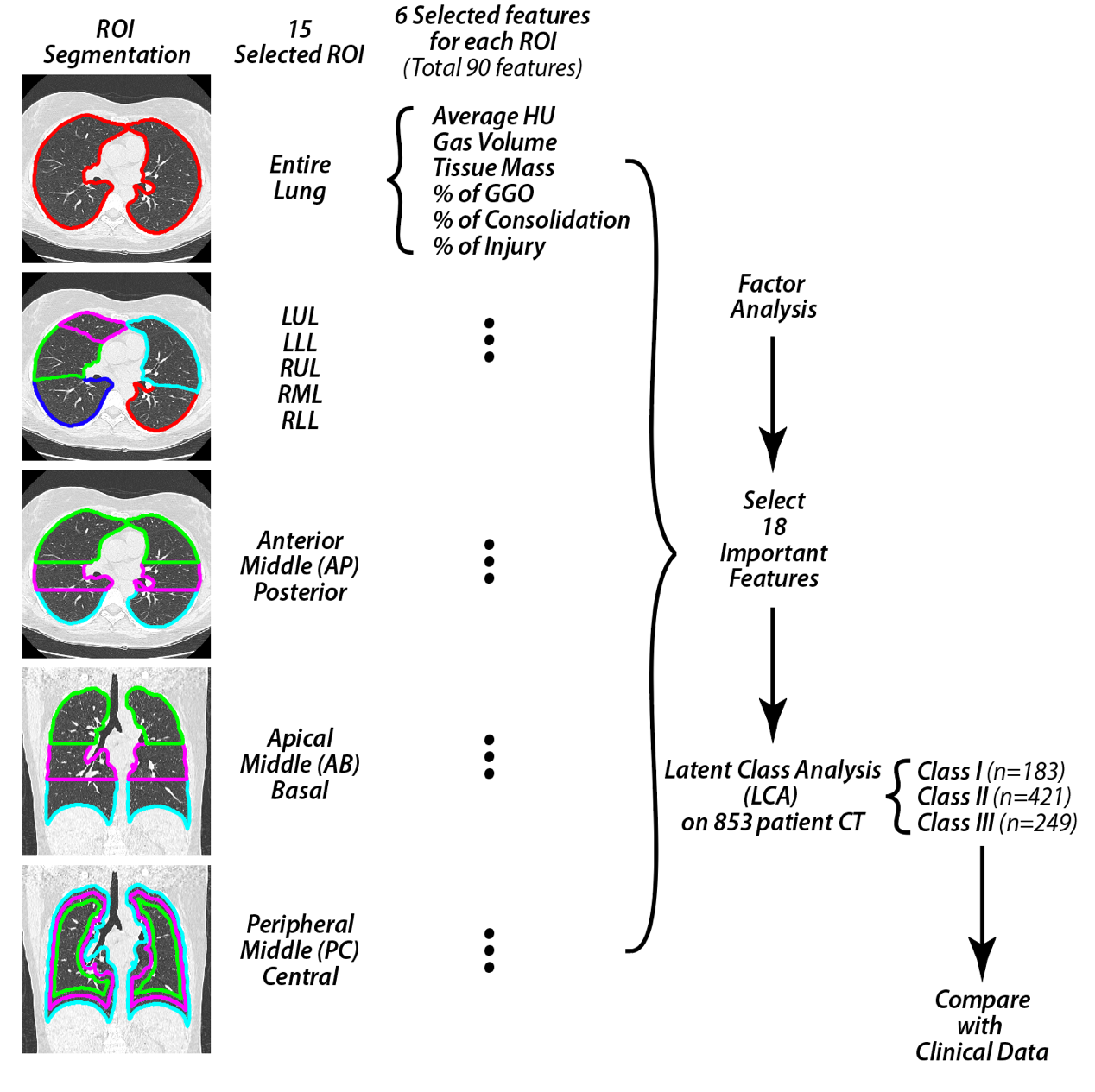** |  |  |  |  |
| **Dorsal** | -641 (148) | -634 (144) | -656 (155) | 0.055 |
| Mean lung density (HU) | 0.9 (0.44) | 0.88 (0.43) | 0.94 (0.46) | 0.059 |
| Lung gas volume (L) | 0.45 (0.17) | 0.45 (0.16) | 0.44 (0.18) | 0.265 |
| Lung weight (kg) | 0.52 (0.23) | 0.54 (0.23) | 0.48 (0.24) | 0.002 |
| Total injury (fraction) | 0.42 (0.17) | 0.43 (0.18) | 0.38 (0.17) | <0.001 |
| GGO (fraction) | 0.1 (0.12) | 0.11 (0.11) | 0.1 (0.12) | 0.616 |
| Consolidation (fraction) | -641 (148) | -634 (144) | -656 (155) | 0.055 |
| **Dorso-Ventral** |  |  |  |  |
| Mean lung density (HU) | -704 (108) | -699 (107) | -716 (109) | 0.034 |
| Lung gas volume (L) | 0.99 (0.42) | 0.97 (0.41) | 1.03 (0.43) | 0.061 |
| Lung weight (kg) | 0.37 (0.11) | 0.38 (0.11) | 0.37 (0.12) | 0.283 |
| Total injury (fraction) | 0.42 (0.2) | 0.43 (0.2) | 0.38 (0.2) | 0.001 |
| GGO (fraction) | 0.35 (0.17) | 0.36 (0.17) | 0.32 (0.16) | <0.001 |
| Consolidation (fraction) | 0.07 (0.06) | 0.07 (0.06) | 0.07 (0.06) | 0.626 |
| **Ventral** |  |  |  |  |
| Mean lung density (HU) | -776 (88) | -773 (86) | -784 (92) | 0.096 |
| Lung gas volume (L) | 1.08 (0.43) | 1.06 (0.42) | 1.12 (0.44) | 0.082 |
| Lung weight (kg) | 0.28 (0.09) | 0.29 (0.09) | 0.28 (0.09) | 0.361 |
| Total injury (fraction) | 0.3 (0.18) | 0.31 (0.18) | 0.28 (0.18) | 0.006 |
| GGO (fraction) | 0.27 (0.16) | 0.29 (0.16) | 0.25 (0.15) | <0.001 |
| Consolidation (fraction) | 0.03 (0.04) | 0.03 (0.03) | 0.03 (0.05) | 0.230 |
| **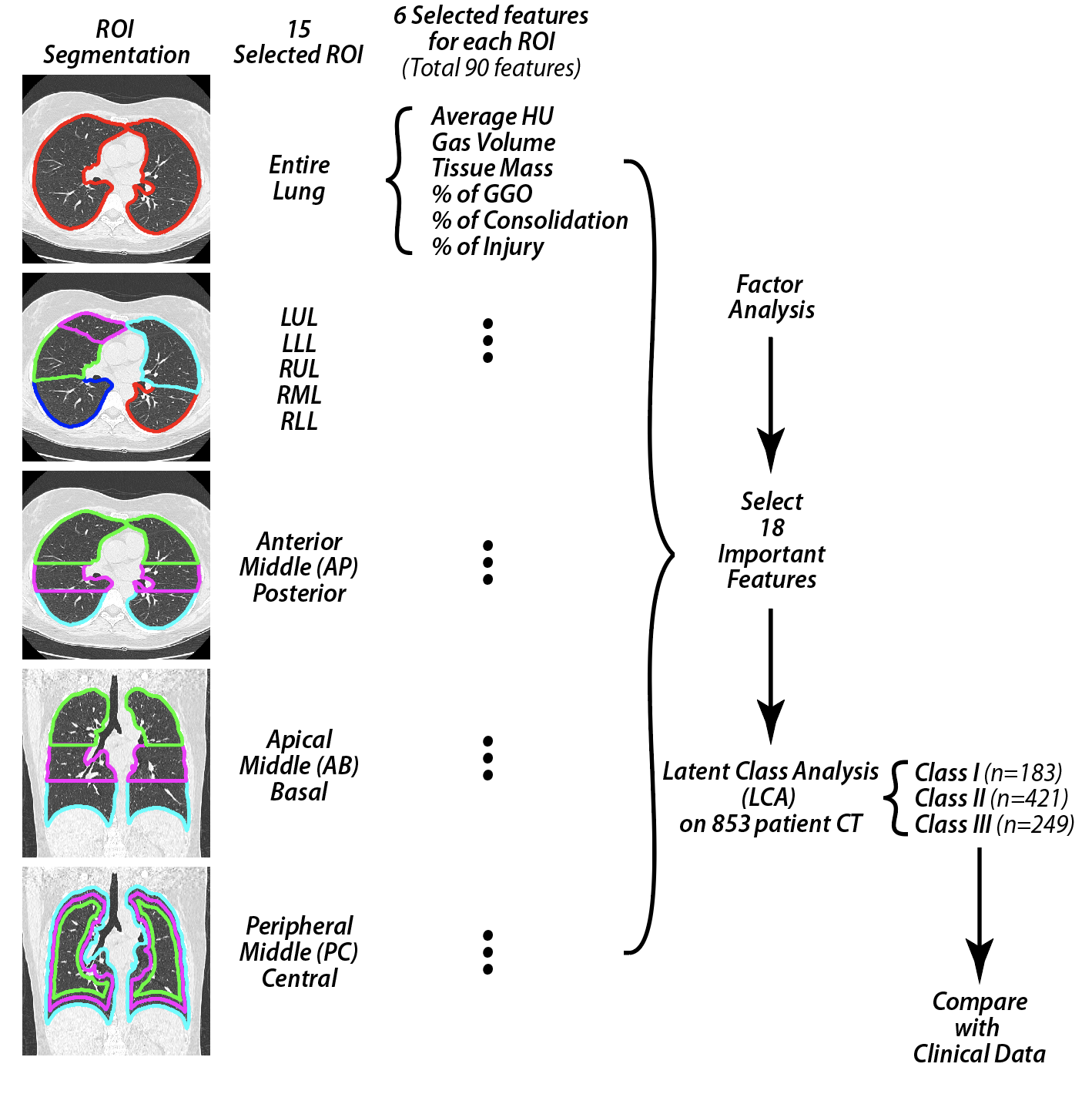** |  |  |  |  |
| **Submantellar** |  |  |  |  |
| Mean lung density (HU) | -691 (107) | -686 (104) | -703 (111) | 0.039 |
| Lung gas volume (L) | 1.06 (0.49) | 1.07 (0.51) | 1.04 (0.43) | 0.319 |
| Lung weight (kg) | 0.45 (0.18) | 0.46 (0.17) | 0.42 (0.19) | <0.001 |
| Total injury (fraction) | 0.45 (0.19) | 0.47 (0.18) | 0.42 (0.19) | 0.002 |
| GGO (fraction) | 0.38 (0.15) | 0.4 (0.15) | 0.35 (0.14) | <0.001 |
| Consolidation (fraction) | 0.07 (0.06) | 0.07 (0.06) | 0.07 (0.07) | 0.938 |
| **Central** |  |  |  |  |
| Mean lung density (HU) | -719 (106) | -715 (104) | -730 (111) | 0.056 |
| Lung gas volume (L) | 0.78 (0.29) | 0.78 (0.29) | 0.78 (0.3) | 0.766 |
| Lung weight (kg) | 0.28 (0.1) | 0.29 (0.1) | 0.27 (0.11) | 0.002 |
| Total injury (fraction) | 0.39 (0.19) | 0.4 (0.19) | 0.36 (0.19) | 0.003 |
| GGO (fraction) | 0.33 (0.16) | 0.34 (0.16) | 0.3 (0.15) | <0.001 |
| Consolidation (fraction) | 0.06 (0.06) | 0.06 (0.06) | 0.06 (0.07) | 0.731 |
| **Hilar** |  |  |  |  |
| Mean lung density (HU) | -718 (115) | -712 (113) | -731 (116) | 0.028 |
| Lung gas volume (L) | 1.13 (0.73) | 1.06 (0.67) | 1.3 (0.81) | <0.001 |
| Lung weight (kg) | 0.37 (0.16) | 0.36 (0.16) | 0.4 (0.18) | 0.002 |
| Total injury (fraction) | 0.39 (0.21) | 0.4 (0.21) | 0.35 (0.21) | <0.001 |
| GGO (fraction) | 0.32 (0.17) | 0.34 (0.17) | 0.29 (0.16) | <0.001 |
| Consolidation (fraction) | 0.06 (0.07) | 0.06 (0.07) | 0.06 (0.07) | 0.573 |

Differences between the 2 clusters of lung CT images were assessed and reported in p-value column. HU=Hounsfield units; GGO: ground-glass opacities. Values are expressed as mean (standard deviation).**Supplemental Figures**

**Supplemental Figure 1. Matrix of pairwise correlations.**

**GGO=ground glass opacities fraction;** PaO_2_/FiO_2_: ratio of arterial oxygen partial pressure to inspiratory oxygen fraction; PaCO_2_: arterial carbon dioxide partial pressure; CRP = C-reactive protein.

**Supplemental Figure 2. Settings of covariance-variance structure.**

**
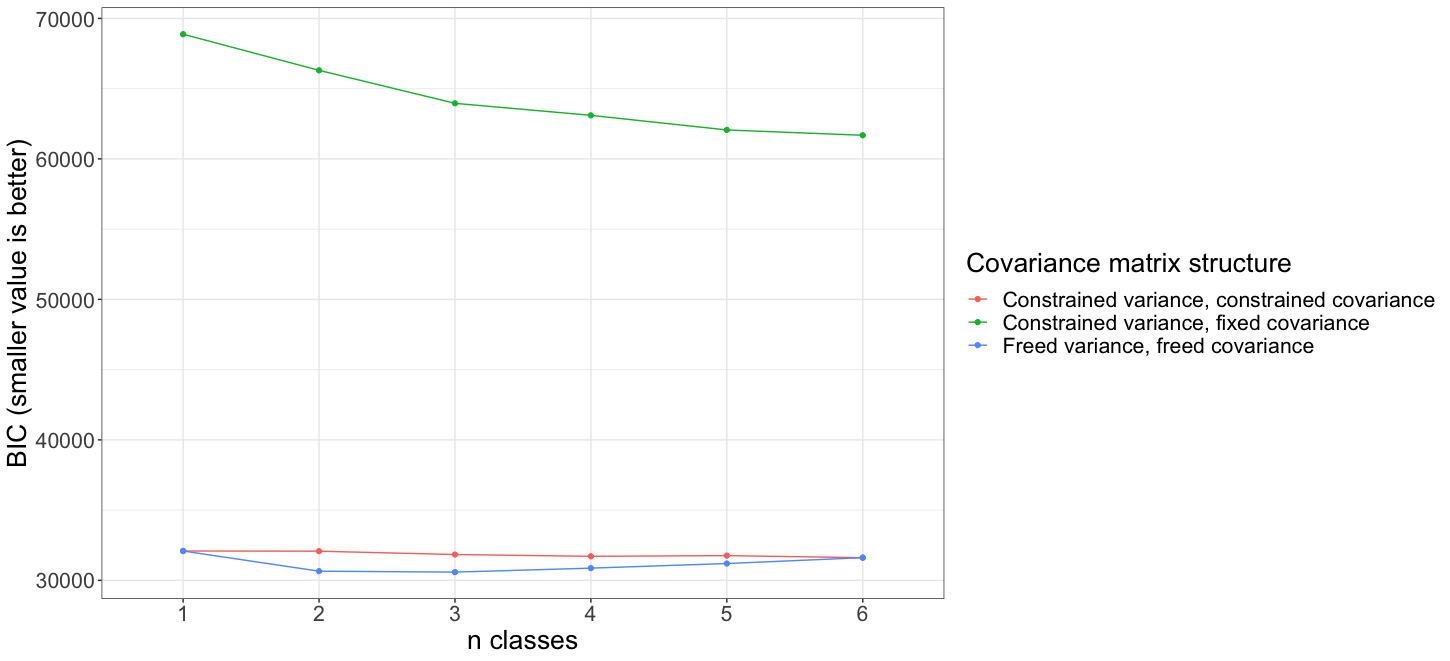
** From red to blue, the models become less constrained (more free). It appears that a two or three-class model with freely-estimated residual variances and covariances fit best (based on interpreting the BIC). However, we also compared the uncertainty from the two or three-class model, and the average uncertainty of three-class model is more than double of two-class model (**Supplemental Table 1**). BIC= Bayesian Information Criterion.

**Supplemental Figure 3.** Study flowchart.


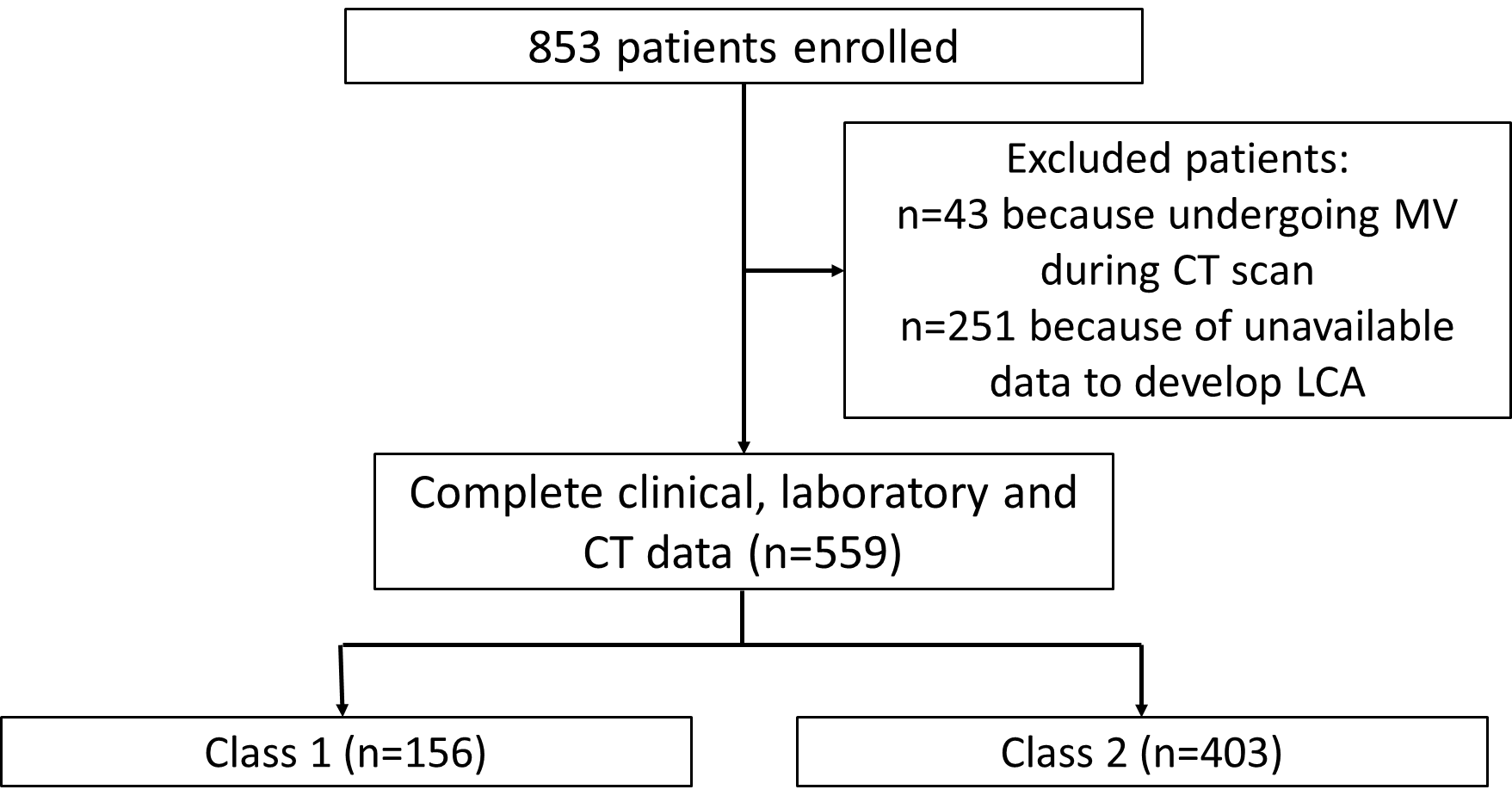


LCA: latent class analysis; CT: computerized tomography; MV: mechanical ventilation.
